# Hepatic immune environment differences among common mouse strains in models of MASH and liver cancer

**DOI:** 10.1016/j.jhepr.2025.101380

**Published:** 2025-03-01

**Authors:** Patrick Huang, Francisco J. Rodriguez-Matos, Jonathan Qi, Rajiv Trehan, Yuta Myojin, Xiao Bin Zhu, Tim F. Greten, Chi Ma

**Affiliations:** 1 Gastrointestinal Malignancy Section, Thoracic and Gastrointestinal Malignancies Branch, Center for Cancer Research, National Cancer Institute, National Institutes of Health, Bethesda, MD, USA; 2 NCI CCR Liver Cancer Program, National Institutes of Health, Bethesda, MD, USA

**Keywords:** Mouse strain, Immune regulation, Liver cancer, Metabolic dysfunction-associated steatohepatitis

## Abstract

**Background & Aims:**

Inbred mouse strains are critical tools for studying immune regulation of metabolic dysfunction-associated steatohepatitis (MASH) and hepatocellular carcinoma (HCC). Here, we profiled mouse strain-associated hepatic immune differences, and performed mice–human cross-species immune comparisons.

**Methods:**

Immune landscapes of C57BL/6, BALB/c, and FVB/N mice were compared under healthy, MASH, or HCC state using high-dimensional spectral flow cytometry (n = 4 per condition). MASH was induced by feeding methionine- and choline-deficient or Western diet + carbon tetrachloride. HCC was caused by hydrodynamic plasmid injection of MYC/sg-p53. Public mouse and human scRNA-seq datasets were used for validation and cross-species comparisons.

**Results:**

In healthy mice, liver CD4^+^ T (24% *vs.* 14% *vs.* 34%, *p* <0.05) and B cells (36.5% *vs.* 35% *vs.*18%, *p* <0.05) varied the most among three strains. C57BL/6 mice showed T_H_1 dominance, whereas BALB/c and FVB/N mice had T_H_2 dominance (log[T_H_1:T_H_2] = 0.17, -0.31, -0.17). In MASH mice, expansion of liver myeloid cells and innate lymphocytes were commonly found, but changes of B cells (log(fold-change) = -0.38, -0.28, -0.58, *p* <0.05) and T subsets (*e.g.* CD4^+^ T log(fold-change) = -0.21, -0.07, -0.15, *p* <0.05) varied greatly among strains. MYC/sg-p53 HCC induced a consistent expansion of liver Tregs and CD8^+^ T cells (*p* <0.05), but differential shifts of liver immune landscape were seen among strains. The flow cytometry data was supported by public scRNA-seq datasets matching C57BL/6 background. Further cross-species comparison in MASH condition confirmed shared changes of adaptive lymphocytes between mice and humans. In two MASH models, BALB/c or C57BL/6 mice were more consistent to recapture loss of CD4^+^ T or B cells, respectively (*p* <0.05).

**Conclusions:**

Substantial liver immune differences exist among common mouse strains. Mice can recapitulate certain human liver immune changes with strain variations.

**Impact and implications:**

Our immune cell profiling study revealed that the liver immune environment can be quite different among common mouse strains both under healthy and pathologic states, such as steatohepatitis or neoplastic processes. Our results serve as a data resource for studies investigating liver immunology and provide valuable insights for the design of studies on various immune cells in the livers of mice.

## Highlights

• High-dimensional flow cytometry-based liver immune profiling of three common laboratory mouse strains.
• Both adaptive and innate immune subsets show substantial variations in mouse strains under baseline, MASH, or HCC conditions.
• Shared changes of adaptive lymphocytes in MASH were found between mice and humans.
• BALB/c or C57BL/6 mice more consistently mimic human loss of CD4+ T or B cells, respectively.

## Graphical abstract

**Figure:**
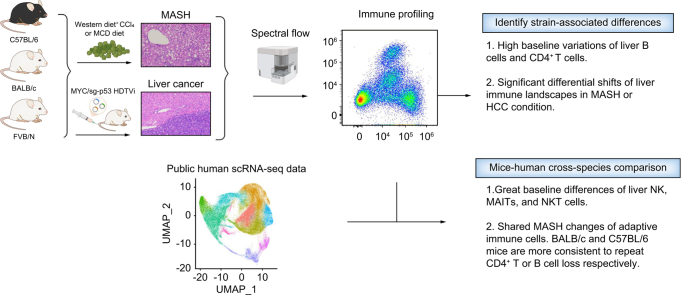

## Introduction

Hepatocellular carcinoma (HCC) is a leading cause of cancer-related deaths, affecting close to 900,000 patients worldwide in 2020.^1^ Aside from having no specific clinical presentation that leads to late-stage diagnoses, the pathophysiology of liver cancer is mainly driven by complex genetic and inflammatory processes that are yet to be fully elucidated.^2^ Metabolic dysfunction-associated steatohepatitis (MASH), a common condition in patients with obesity, is a rising risk factor for liver cancer following the global obesity epidemic.^3^ Mice have been used in immunology research for years to get a better understanding of cancer immunology because of their capability to mount adequate immune responses against cancerous tissues. To study liver cancer, multiple mouse models have been established, and the optimal use of these models has been described. For some models, specific mouse strains are required to adequately study biologically relevant cell populations or induce the disease of interest.^4^ However, multiple mouse strains can be used to induce liver cancer by novel techniques of genetic engineering.^5^^,^^6^

Immune responses vary between strains of mice, as reported in previous studies. Specifically, mice of C57BL/6 and BALB/c strains have been well characterized for years as having T_H_1 and T_H_2 dominant immune responses, respectively, defined by their differences in expression of interferon γ (IFNγ) or IL-4.^7^ Aside from immune responses, recent studies have demonstrated that different strains of mice express varying baseline immune cell compositions in their systems. For example, a study published by Petkova *et al.*^8^ showed that peripheral blood leukocyte subset proportions differed among mature mice (>6 months old) from different strains, and among sexes of these mice. Another study highlighted that differences also exist among strains of mice and between sexes in the immune cell composition of bone marrow and spleen.^8^ However, there are no studies that report if these differences are also present in mouse livers among strains.

In this study, we highlight immune cell population differences in the livers within three mouse strains commonly used for cancer and immunology research. Moreover, we highlight how these populations change in different pathological conditions. We provide a comprehensive analysis of major and less abundant immune cell subsets commonly studied in the context liver immunology. In addition, we describe and analyze these differences in spleens to depict relevant changes in this secondary lymphoid organ upon pathologic state induction. With this study, we depict the immune landscape between strains of mice, with the goal of limiting confounders in future studies within cancer research and establishing appropriate animal models for the study of liver immunology in cancer and beyond.

## Materials and methods

### Animal handling

All animal protocols followed were developed in accordance with the PHS Policy on Humane Care and Use of Laboratory Animals. These were submitted and approved by the NIH before being performed. For this study, we used 6–8-week-old BALB/c, C57BL/6, and FVBN strain mice, obtained from Charles River (Frederick, MD, USA; Strain codes 027, 028, and 207). Mice were kept in isolation cages, at a maximum of five mice per cage. These were placed under conditions of stable temperature, humidity, and 12-h light cycle periods in accordance with their circadian rhythm. Mouse sex is specified in the figure legends for each experiment.

### Diet

Regular chow diet was administered to mice from control groups, as well as to those who received plasmid vectors for liver cancer induction. MASH was induced in mice by feeding a methionine- and choline-deficient (MCD) diet (Research diets inc., New Brunswick, NJ, USA Ref. A02082002BR) for a period of 3 weeks,^9^ or a Western diet (Envigo, Indianapolis, IN, USA, Ref. TD.120528) with high sugar solution (23.1 g/L d-fructose and 18.9 g/L d-glucose) with weekly intraperitoneal injections of carbon tetrachloride (CCl_4_) (Sigma, St. Louis, MO, USA, Cat# 289116) at a dose of 0.32 mg/g of body weight for 12 weeks as previously reported.^10^

### Hydrodynamic tail vein injection

Previously described plasmid vectors^11^ were administered in 1.7–2.0 ml of PBS solution, in a period of 1–2 s. Each mouse received 30 μg pT3-MYC-Luc (MYC), 10 μg px330-TP53-Cas9 (sg-p53), and 2.5 μg SB13 transposase vectors. Mice were humanely sacrificed 3–4 weeks following injections. Final decision on harvest was based upon the clinical progress of mice in this time.

### Flow cytometry

Both livers and spleens underwent mechanical dissociation, with livers undergoing additional purification with Isotonic Percoll (diluted in PBS) obtained from Cytiva (Marlborough, MA, USA). ACK Lysis Buffer, obtained from Quality Biological (Gaithersburg, MD, USA) was used on all samples for red blood cell exclusion and leukocyte optimization. Samples were resuspended in flow cytometry stain buffer for analysis. Staining was performed using antibodies listed in the Supplementary CTAT method tables. Samples were measured using Cytek Aurora 5L (Fremont, CA, USA) and analyzed with FlowJo software (v10.9.0). Gating strategies employed for myeloid and lymphoid cell lineages are shown in (Fig. S1A–C).

### scRNA-seq data analysis

Mouse scRNA-seq datasets GSE231712 and GSE156059, and the human scRNA-seq dataset GSE159977 were used. Datasets were processed using the Seurat package (v5.1.0). Cells with low sequencing quality (nFeature_RNA <500 or >4,000; nCount_RNA <500 or >14,000; mitochondrial gene percentage >10, log_10_GenesPerUMI <0.8) were removed. After data normalization, 2,000 highly variable genes were identified, which were followed by data scaling and principal component analysis-based dimensionality reduction. The uniform manifold approximation and projection (UMAP) was generated by using the first 30 principal components. Harmony (v1.2.3) was used for batch correction for human dataset. Cell clusters were identified by the FindClusters function at varied resolutions from 0.1 to 0.8 following the FindNeighbors function. Annotation of major clusters was based on both marker genes identified by the FindMarker function and verification by the SingleR package (v2.1.2) using the ImmGenData or HumanPrimaryCellAtlasData database for mouse or human cells, respectively. The annotation of T cells in the human dataset GSE159977 was based on the original publication.^12^ Annotation of the further subclustered immune cells following the Subset function was performed manually with several rounds of marker gene identification from clusters segregated at various resolutions.

### Statistical analysis

Data was analyzed using PRISM Graph Pad (v10.2.0 (355), GraphPad Software, San Diego, CA, USA). Two-way ANOVA was used for comparison of cell differences between strains, following grouped analysis and corrections with Tukey’s multiple comparisons test. These results were plotted as bar graphs, with representation of mean and SEM. Analysis of multiple unpaired *t* tests with no correction for multiple comparisons was used to compare cell differences between pathologic and control mice among the same strain. Data was presented as superimposed bar graphs, highlighting the differences of the cell means of each mice strain in regular *vs.* in pathologic states. Also, data regarding the comparison between control and pathologic mice were exported to Excel (Microsoft Corp, Redmond, WA, USA) where log_2_ fold changes, *t* tests, standard deviations, and Z-scores were calculated. The data were placed onto Biowulf (NIH HPC Linux cluster) and downstream analyses was performed under RStudio (version 2023.09.1+494, RStudio, Inc. Boston, MA, USA). Data were plotted with the ggplot2 package (version 3.5.0).

## Results

### Baseline liver immune profile variations among mouse strains

Baseline liver immune cell profiles of three commonly used mouse strains, C57BL/6, FVB/N, and BALB/c were characterized using high-dimensional spectral flow cytometry. Splenic immune cells were included in the analysis to help identify whether the strain-specific immune differences were limited to the liver. To facilitate overviewing the immune landscape, CD45^+^ immune cells were first categorized based on lineage and abundance into five major lymphoid subsets (CD4^+^ T cells, CD8^+^ T cells, innate-like T cells, B cells, and innate lymphoid cells) and myeloid cells. The remaining cells were categorized as unidentified cells. Then the major immune subsets were further separated as following. CD4^+^ T cells were further sub-grouped into regulatory T cells (Tregs), T_H_1, and T_H_2 based on transcriptional factor expression. Innate-like T cells (ILTCs) were subdivided into invariant natural killer T (iNKT) cells, mucosal-associated invariant T (MAIT), and γδT cells. Innate lymphoid cells (ILCs) were grouped into natural killer (NK), ILC1, ILC2, and ILC3. Myeloid cells were separated into dendritic cells, neutrophils, and macrophages. The gating strategy used is shown in Fig. S1A–C. It should be noted that our liver immune cell isolation method was optimized for lymphoid cell recovery, which comprised ∼90% of the total isolated liver CD45^+^ cells. Although being substantially present in the liver microenvironment, Kupffer cells were rare in this study as optimal recovery requires liver perfusion, collagenase digestion, and avoidance of Percoll gradient centrifugation.

**Fig. 1:**
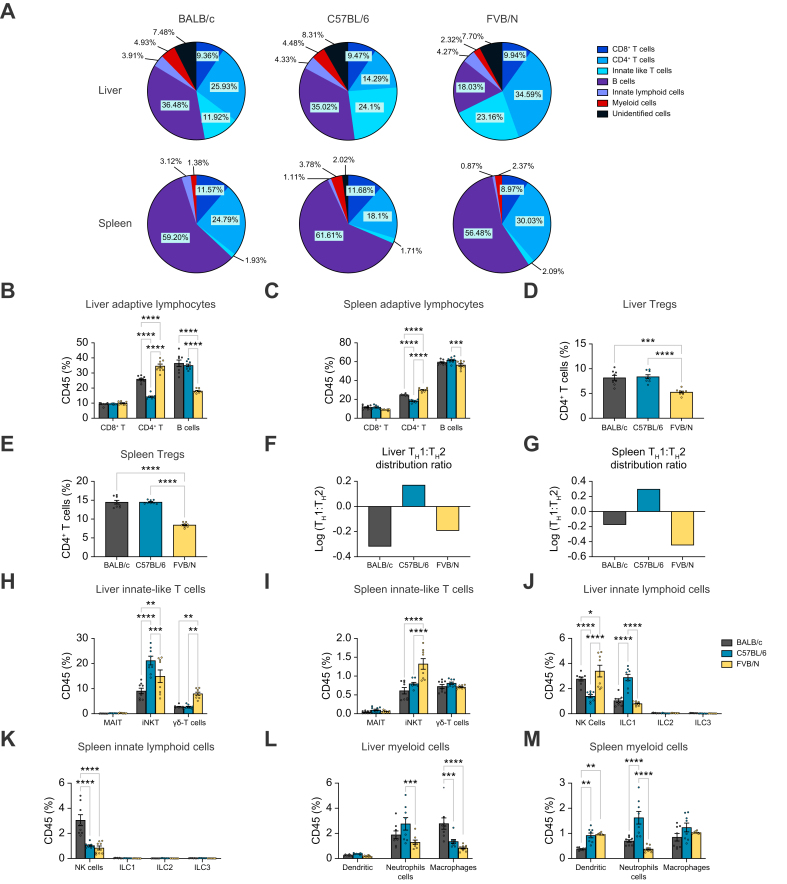
Profiling baseline immune subsets in liver and spleen of BALB/c, C57BL/6 and FVB/N mice. Liver and spleen immune cells were prepared from female naïve BALB/c, C57BL/6, and FVB/N mice, and then proceeded to high-dimensional flow cytometry analysis. The frequencies of each immune subsets in total CD45^+^ cells were calculated using FlowJo. (A) The composition of liver or spleen CD45^+^ immune cells are shown at the level of major immune subsets in three mouse strains. The major immune subsets include CD8^+^ T cells, CD4^+^ T cells, innate-like T cells, B cells, innate lymphoid cells, and myeloid cells. (B–M) The levels of individual immune subsets from liver or spleen were compared among three mouse strains. The subsets were grouped into adaptive lymphocytes including CD8^+^ T cells, CD4^+^ T cells and B cells (B,C), T helper subsets including Tregs, Th1 and Th2 (D-G), innate-like T cells including MAIT, iNKT, and γδT cells (H, I), innate lymphoid cells including NK and ILC1/2/3 (J, K), and myeloid cells including dendritic cells, neutrophils, and macrophages (L, M). n = 8 per group, two-way ANOVA with Bonferroni correction, ∗*p* <0.05; ∗∗*p* <0.01; ∗∗∗*p* <0.001; ∗∗∗∗*p* <0.0001. Tregs, regulatory T cells.

In all the three mouse strains, the liver immune cells were dominated by T cell subsets, ranging from ∼45% (BALB/c and C57BL/6) to ∼66% (FVB/N) of total CD45^+^ cells (Fig. 1A). These subsets were comprised of CD4^+^ T cells, CD8^+^ T cells and ILTCs. The level of liver CD8^+^ T cells (∼10%) was fairly stable among mouse strains (Fig. 1A and B). In contrast, liver CD4^+^ T cell levels varied, and represented the most abundant liver immune cell type in FVB/N mice (∼24% in BALB/c, ∼14% in C57BL/6, and ∼34% in FVB/N) (Fig. 1A and B). A prominent population of ILTCs was found in the liver with lower levels in BALB/c mice compared with C57BL/6 and FVB/N mice (∼10% in BALB/c, ∼20% in C57BL/6, and FVB/N) (Fig. 1A). Besides T cells, mouse livers harbored large but varying numbers of B cells depending on the specific mouse strain. B cells represented the most abundant liver immune cells in both BALB/c and C57BL/6 mice (∼36.5% and ∼35% respectively), but only accounted for 18% of the liver immune cells in FVB/N mice (Fig. 1A and B). The levels of liver ILCs were similar (∼4%) among all three strains (Fig. 1A). Myeloid cells comprise less than 5% of liver CD45^+^ cells, where their levels were similar (∼4%) between BALB/c and C57BL/6 mice and lower in FVB/N mice (∼2%) (Fig. 1A).

Unlike in the liver, B cells were the most abundant immune cells present in the spleens (Fig. 1A). The three adaptive immune cells (B cells, CD4^+^ T cells, and CD8^+^ T cells) consistently made up >90% of total splenic CD45^+^ cells. Although FVB/N mice had significantly lower splenic B cell composition (∼56%) compared with BALB/c (∼59%) and C57BL/6 (∼61%), differences in B cell populations between strains were much smaller, compared with those in the liver (Fig.1A and C). Interestingly, splenic CD8^+^ T cells presented with similar levels (∼10%) as in the liver and remained stable across mouse strains (Fig. 1A and C). Splenic CD4^+^ T cells varied among mouse strains (∼24% in BALB/c, ∼18% in C57BL/6, ∼30% in FVB/N), but followed a pattern similar to that seen in liver CD4^+^ T cells (Fig. 1A and C). This resulted in variations in splenic CD4^+^ T to CD8^+^ T cell ratios between mouse strains, but these remained similar between hepatic and splenic tissues from the same mouse strain, suggesting a systemic regulation. Substantially lower levels of ILTCs (∼2%) were found in spleen compared with the liver (Fig. 1A).

Based on the expression of transcription factors, CD4^+^ T cell subsets including FOXP3^+^ Tregs, T-bet^+^ T_H_1 cells, and GATA3^+^ T_H_2 cells were identified. FVB/N mice had the highest level of hepatic CD4^+^ T cells but had the lowest level of hepatic Treg composition among CD4^+^ T cells (5.3% of CD4^+^ T cells) when compared with C57BL/6 (8.4% of CD4^+^ T cells) and BALB/c mice (8.3% of CD4^+^ T cells) (Fig. 1D). Although higher levels of Tregs were found in spleens in general, the relative levels of splenic Tregs among mouse strains followed the same pattern as hepatic Tregs (Fig. 1E). Additionally, the ratios of T_H_1:T_H_2 were calculated to indicate the T helper cell functional status, and the data were depicted as logarithmic transformation. BALB/c and FVB/N mice livers had a baseline predominance of T_H_2 cells in comparison with T_H_1 (log(T_H_1:T_H_2) = -0.32 and -0.19, respectively), and conversely, C57BL/6 had a predominance of T_H_1 cells (log[T_H_1:T_H_2] = 0.17) (Fig. 1F). Similar results were found in spleens (Fig. 1G). These results are consistent with the well-documented T_H_1 dominant responses in C57BL/6 mice while T_H_2 dominant responses were present in BALB/c mice.

The ILTCs were further studied and separated into iNKT, γδT, and MAIT cells. Although total liver ILTC levels were similar between C57BL/6 and FVB/N mice (Fig. 1A), C57BL/6 livers had more hepatic iNKT cells (21.2% in C57BL/6 *vs.* 14.9% in FVB/N) but fewer γδT cells (2.73% in C57BL/6 *vs.* 7.97% in FVB/N) (Fig. 1H). BALB/c livers had the lowest frequencies of iNKT (9.1%) compared with the other two strains, and a similar level of γδT cells (2.76%) compared with C57BL/6 (Fig. 1H). The ILTCs were much less abundant in spleens (∼2%) (Fig. 1A). The highest splenic iNKT cell populations were found in FVB/N (1.32%) followed by C57BL/6 (0.8%) and BALB/c mice (0.6%) (Fig. 1I). No difference in hepatic γδT cells were found among mouse strains. Consistent with the reported low level of MAIT cells in mice, minimal MAIT cells were detected in all three mouse strains included in this study (Fig.1I).

ILCs were sub-grouped into NK cells, ILC1, ILC2, and ILC3. NKp46 was used in BALB/c mice to help identify NK cells because of the lack of NK1.1 expression in these mice. Liver NK and ILC1 were separated based on CD49b or CD49a expression, respectively. The gating strategy used is shown in detail in Fig. S1B. Hepatic ILCs mainly comprised NK cells and ILC1s, whereas ILC2s and ILC3s were detected at minimal levels (Fig. 1J). Although the levels of total liver ILCs were similar among mouse stains, C57BL/6 mice had significantly reduced NK cells but increased ILCs1 in comparison with its strain counterparts (Fig. 1A and J). Unlike in the liver, splenic ILCs mainly contained NK cells, and both C57BL/6 and FVB/N mice showed lower NK cell levels compared with BALB/c mice (Fig. 1K).

Myeloid cell subsets including neutrophils, macrophages, and dendritic cells were also measured. Significant differences in liver neutrophils (2.8% in C57BL/6, 1.9% in BALB/c, and 1.3% in FVB/N) and macrophages (BALB/c [2.8%], C57BL/6 [1.4%], and FVB/N [0.83%]) were found among mouse strains (Fig. 1L). No differences in liver dendritic cells among strains were observed. Within spleens, the findings in strain-associated variation of neutrophils (1.62% in C57BL/6, 0.7% in BALB/c, 0.38% in FVB/N) were similar to those seen in livers (Fig. 1M). However, in contrast to the hepatic microenvironment, a reduced population of splenic dendritic cells was found in BALB/c mice exclusively (0.97% in FVB/N, 0.92% in C57BL/6, 0.38% in BALB/c) with no significant change in splenic macrophages among mouse strains (Fig. 1M).

In summary, we noticed that CD8^+^ T cell levels were quite stable, but other immune subsets varied to some extent among the three mouse strains included in this study. In the livers, the differences with most significance were observed for CD4^+^ T cells, B cells, and ILTCs. The mouse strain differences in CD8^+^ T cells and CD4^+^ T subsets were found in both the liver and spleen, suggesting a systemic regulation, whereas the strain differences of B cells and ILTCs were mainly limited to the liver.

## Mouse strain-specific pertubations of hepatic immune cells in MASH

**Fig. 2:**
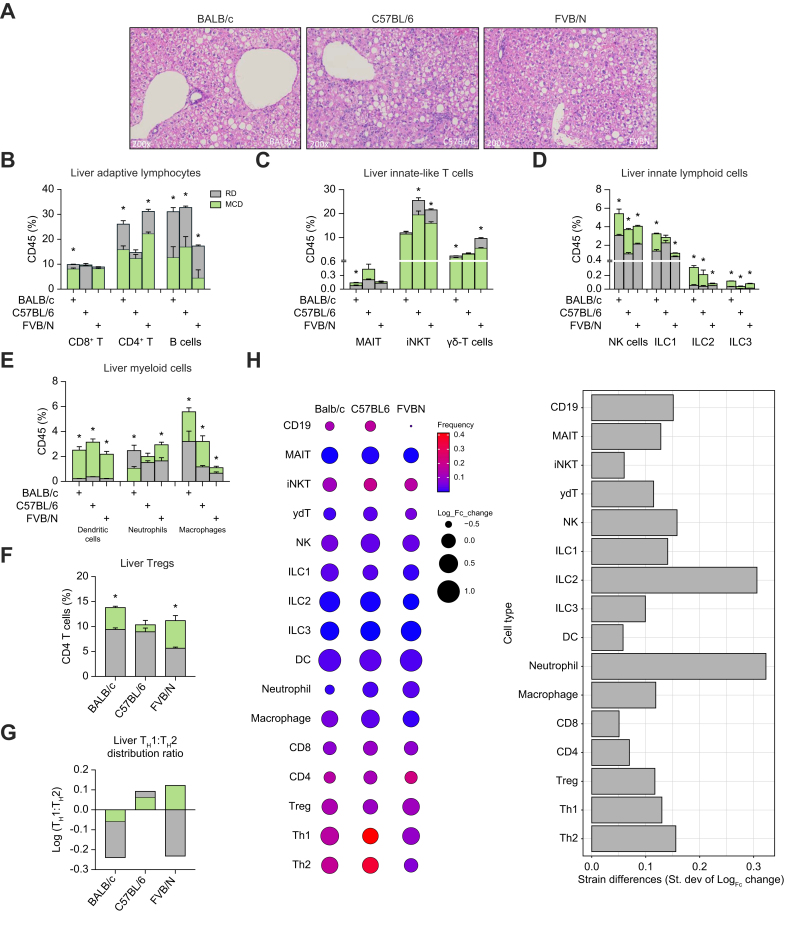
Changes of liver immune subsets under MASH in BALB/c, C57BL/6, and FVB/N mice fed with MCD diet. Female BALB/c, C57BL/6, and FVB/N mice were fed with MCD diet (*vs.* regular diet) to induce MASH. (A) The development of MASH was confirmed by H&E staining. Liver immune cells from MASH mice or control mice were prepared and immune subsets were measured by flow cytometry analysis. The comparison between MCD (green) and control (gray) was performed in each liver immune subsets of the three mouse strains (B–G). The overall changes of various liver immune subsets from three mouse strains are shown (H). The size of circle represents the log_10_ transformed fold changes of each immune subset. The color gradient represents the relative frequences of each immune subset. The distribution of fold changes of each type of immune cell is also shown; n = 4 per group, two-way ANOVA with Bonferroni correction, ∗*p* <0.05. MASH, metabolic dysfunction-associated steatohepatitis; MCD diet, methionine- and choline-deficient diet; Tregs, regulatory T cells.

MASH is a rising risk factor for HCC, and multiple reports suggest that the immune system has a critical role in MASH progression.^3^^,^^13^ Therefore, we tested the potential impact of mouse strain usage for investigating immune regulations by MASH. BALB/c, C57BL/6, and FVB/N mice were kept on an MCD diet for a total of 3 weeks to induce MASH, or regular diet to serve as control. Histologic analysis revealed similar micro- and macrovesicular steatosis within the liver parenchyma across mouse strains, confirming the establishment of MASH (Fig. 2A). Liver immune cells were identified and analyzed using flow cytometry. Under a MASH state, liver immune cells comprised the same major immune subsets as described above, still dominated by T cell subsets (Fig. 2B, Fig. S2A–F). However, substantial changes were encountered when compared with healthy controls, and these changes varied among mouse strains. When compared with controls, MASH caused broad decreases in liver T and B cell subsets and conversely resulted in increases among both ILCs and myeloid cells across mouse strains (Fig. 2B–E). One of the most striking findings was an observed decrease of >50% of liver B cells in all the mouse strains when compared with controls (Fig. 2B), especially considering its abundant baseline levels. The hepatic B cell reduction was comparable between BALB/c and C57BL/6 mice but was even more drastic in FVB/N mice (∼75%) which had the lowest B cells at baseline (Fig. 2B). Marked liver CD4^+^ T cell loss (∼30% to 40%) was observed in BALB/c and FVB/N mice (Fig. 2B). However, only a marginal decrease of CD4^+^ T cells was found in C57BL/6 mice which had the lowest baseline level of the three mouse strains used in this study (Fig. 2B). In contrast to B cells or CD4^+^ T cells, CD8^+^ T cells remained stable (∼10% change) in MASH mouse livers except for a significant but small drop that was seen among BALB/c mice (Fig. 2B). The changes in liver ILTCs were moderate compared with B cells or CD4^+^ T cells, but expressed greater variations between mouse strains (Fig. 2C). With great reductions of B cells and CD4^+^ T cells, ILTCs became more prominent in MASH liver immune cells. A 20–25% reduction of hepatic iNKT cells was seen in C57BL/6 and FVB/N mice with MASH, but liver iNKT levels did not change in BALB/c mice (Fig. 2C). More γδT cells were found in the livers of BALB/c and FVB/N mice with MASH, but no change was seen in C57BL/6 mice of this model (Fig. 2C). A widespread increase in hepatic ILCs was observed in all three mouse strains with MASH (Fig. 2D). Hepatic NK cell/ILC1 levels were more than doubled, and ILC2 and ILC3 increased by 1.5–5 times (Fig. 2D). Significant increases of myeloid cells, particularly among liver dendritic cells, were also identified (Fig. 2E). Liver neutrophils increased in C57BL/6 and FVB/N but decreased in BALB/c mice. MASH increased liver macrophage levels in all three mouse strains and the effect was stronger in C57BL/6 and BALB/c mice compared with FVB/N mice.

Upon induction of MASH, FOXP3+ Tregs occupied a higher proportion of CD4^+^ T cells among BALB/c mice (13.8%) when compared with C57BL/6 mice (10.4%) (Fig. 2F, Fig. S2E). Moreover, staying consistent with strain-specific immune responses previously established, C57BL/6 mice presented with a high T_H_1:T_H_2 ratio of CD4^+^ T cells (log[T_H_1:T_H_2] = 0.06), whereas BALB/c mice had a T_H_1:T_H_2 ratio of less than 0 (log[T_H_1:T_H_2] = -0.06) (Fig. 2G, Fig. S2F). Although there are no previous studies establishing this predominance of CD4^+^ T helper cells in FVB/N mice, this study revealed that FVB/N mice display a log[T_H_1:T_H_2] ratio of 0.12 when exposed to an MCD diet for establishment of MASH (Fig. 2G, Fig. S2F). Interestingly, the shifts in T helper cells upon MASH induction were similar among BALB/c and FVB/N strain mice, significantly increasing the proportionality of T_H_1 to T_H_2 cells when compared with controls (log[T_H_1:T_H_2] = -0.06 and 0.12, respectively) (Fig. 2G, Fig. S2F). However, despite the increase in this proportion, T_H_2 cells were still higher among T helper cells in comparison with T_H_1 cells present in BALB/c mice. Additionally, despite a higher increase in T_H_2 cells compared to that seen in T_H_1 cells in a MASH state, C57BL/6 mice maintained a higher T helper cell state in T_H_1 cells (log[T_H_1:T_H_2] = 0.06) (Fig. 2G, Fig. S2F).

Changes in the shifts of immune cells in comparison to controls were presented as a logarithmic transformation and summarized in Fig. 2H. Between mouse strains, the most striking differences in shift of immune cell subsets after induction of MASH were perceived among neutrophils (SD = 0.32), followed by ILC2 (SD = 0.31) and B cells (SD = 0.15). The most impactful increases in the fold-change of immune cells by MASH among the three mouse strains was seen in dendritic cells (logF[c] = 1.03 in BALB/c, 0.97 in FVBN, 0.91 in C57BL/6), followed by ILC2s (logF[c] = 0.74 in BALB/c, 0.70 in C57BL/6, 0.19 in FVB/N) and ILC3s (logF[c] = 0.68 in FVB/N, 0.63 in C57BL/6, 0.49 in BALB/c). Establishment of MASH also provoked a rise in cell composition of hepatic macrophages, NK cells, and MAIT cells among all strains. Aside from these, significant decreases in cell populations were also seen among B cells (logF[c] = -0.58 in FVB/N, -0.39 in BALB/c, -0.28 in C57BL/6), followed by CD4^+^ T cells (logF[c] = -0.21 in BALB/c, -0.15 in FVB/N, -0.07 in C57BL/6) and γδT cells (logF[c] = -0.23 in FVB/N, -0.19 in C57BL/6, -0.02 in BALB/c). Moreover, different shifting patterns were observed on neutrophil populations, as these decreased in BALB/c mice (logF[c] = -0.36) but conversely increased within both C57BL/6 and FVB/N mice (logF[c] = 0.12 and 0.25, respectively). In terms of immune cell shifts when compared with controls, T_H_1 cell proportionality increased significantly among both BALB/c (logF[c] = 0.22) and FVB/N (logF[c] = 0.18) mice. However, shifts of Tregs and T_H_2 cells behaved differently among strains, as BALB/c mice showed a significant increase in T_H_2 cells (logF[c] = 0.05) and a decrease in Tregs (logF[c] = -0.05), whereas FVB/N mice showed a significant increase in Tregs, exclusively (logF[c] = 0.15). Among CD4^+^ T cell subsets, T_H_2 cells presented with the most striking differences in immune cell shifts (SD = 0.16) between the strains included in the study.

Splenic immune cell changes were also evaluated in the context of MASH mice (Fig. S3A–G). Compared to the liver, MASH caused less immune perturbation in the spleen. B cells, CD4^+^ T cells, and CD8^+^ T cells still comprised >90% of total splenic CD45^+^ cells, and MASH did not change the levels of either CD4^+^ T cells or CD8^+^ T cells significantly (Fig. S3A and B). Unlike in the liver, the shifts of B cell levels by MASH were milder on splenic tissues. In contrast to a ∼10% B cell reduction in BALB/c and C57BL/6 mice spleens, FVB/N mice showed a ∼10% B cell increase (Fig. S3A and B). ILTCs remained as a small proportion (<2%) of CD45^+^ cells among splenocytes. Decreases in iNKT cells and increases of MAIT cells were seen similar to the liver (Fig. S3C). An increase in splenic NK cells was also found in all three mouse strains (Fig. S3D). The upregulation of ILCs was less consistent among mouse strains compared with those seen in the liver (Fig. S3D). Additionally, no clear pattern of change was noted on splenic myeloid cells between strains after MASH induction (Fig. S3E).

The impact of MASH on splenic CD4^+^ T cell subsets was also evaluated. Unlike the increase of Tregs seen across strains in the liver, MASH had little effect on the population size of Tregs among splenic immune cells (Fig. S3F). However, similar to the liver, increased T_H_1:T_H_2 ratios were encountered, showing that MASH shifted CD4^+^ T helper cell function towards T_H_1 dominance across all mouse strains (Fig. S3G). The increase in T_H_1 cells was enough to cause a conversion from baseline T_H_2 dominance to T_H_1 dominance in BALB/c spleens.

Comparison of splenic immune cell shifts by MASH between mouse strains was also considered (Fig. S3A). As described above, most of these differences were still in the same direction as the liver but often to a greatly reduced extent. In mice spleens, MASH led to a significant rise in ILC1 (logF[c] = 0.78 in FVB/N, 0.37 in C57BL/6, 0.09 in BALB/c), NK cells (logF[c] = 0.43 in FVB/N, 0.36 in C57BL/6, 0.20 in BALB/c), and MAIT cells (logF[c] = 0.38 in BALB/c, 0.15 in FVB/N, 0.004 in C57BL/6). MASH also caused a decrease among all strains within splenic iNKT cells, although not to equal proportions (logF[c] = -0.29 in BALB/c, -0.20 in C57BL/6, -0.11 in FVB/N). Populations of CD4^+^ T cells, CD8^+^ T cells, γδT cells, and neutrophils were similar within strains upon MASH induction.

Altogether, our study demonstrated that the MCD diet had a stronger immune influence in the liver than in spleens, causing a drastic reduction of liver T cells and B cells, as well as an increase of liver ILCs and myeloid cells. The alterations of T cells and B cells showed great variation among mouse strains. In contrast, the changes in ILCs and myeloid cells were more consistent. Although hepatic CD4^+^ T helper cells showed a consistent shift towards T_H_1 dominance in our MASH model, they could remain at either T_H_1 or T_H_2 dominance function status respective to the specific mouse stain.

**Fig. 3:**
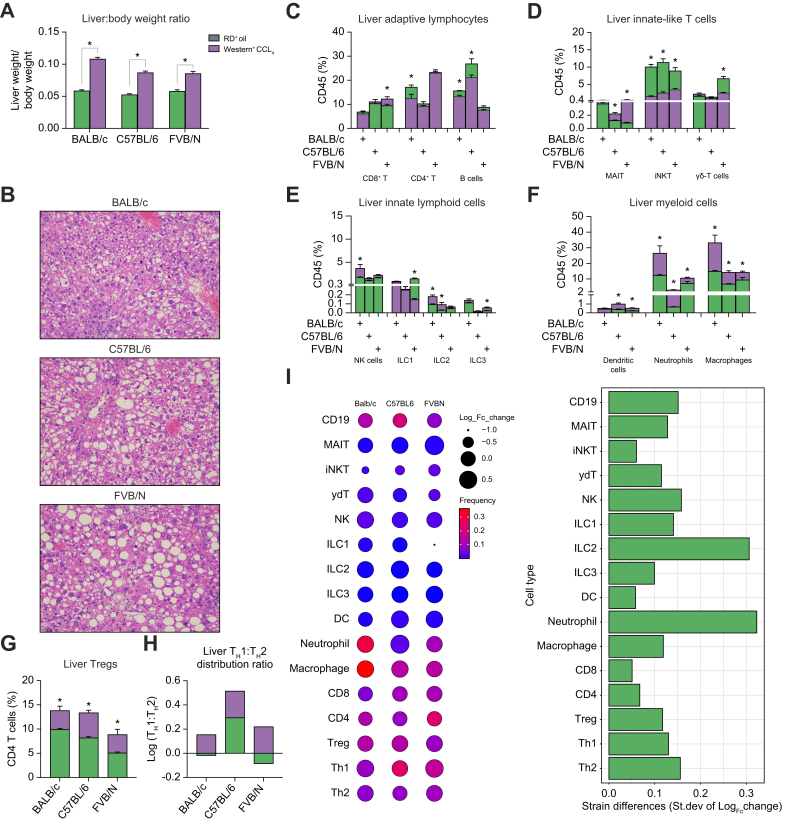
Liver immune cell changes under MASH in BALB/c, C57BL/6, and FVB/N mice treated with Western diet + CCl_4_. Female BALB/c, C57BL/6 and FVB/N mice were kept on a Western diet + CCl_4_ (*vs.* regular diet) to induce MASH. The development of MASH was confirmed by measuring the liver-to-body weight ratio (A) and H&E staining (B). Liver immune cells from MASH mice or control mice were prepared and immune subsets were measured by flow cytometry analysis. The comparison between Western diet + CCl_4_ (brown) and control (gray) was performed in each liver immune subsets of the three mouse strains (C–H). The overall changes of various liver immune subsets from three mouse strains are shown (I). The size of circle represents the log_10_ transformed fold changes of each immune subset. The color gradient represents the relative frequences of each immune subset. The distribution of fold changes of each immune cells is also shown; n = 4 per group, two-way ANOVA with Bonferroni correction, ∗*p* <0.05. CCl_4_, carbon tetrachloride; MASH, metabolic dysfunction-associated steatohepatitis; Tregs, regulatory T cells.

Changes in the composition of hepatic immune cells among mouse strains were further investigated in a second MASH model. Mice were kept on a Western diet and CCl_4_ was injected. This model has previously been reported to closely resemble human MASH.^10^ Consistent with previous reports, the Western diet + CCl_4_ treatment increased the liver-to-body weight ratio in all mice (Fig. 3A), and the development of MASH was confirmed by histology (Fig. 3B). Interestingly, the majority of the MCD diet-induced liver immune changes (Fig. 2B–H) could be recapitulated in the Western diet + CCl_4_ model (Fig. 3C–I), including the decrease of CD4+ T cells, B cells, iNKT cells, γδT cells, as well as increases in MAIT cells, ILC2/3s, dendritic cells, neutrophils, macrophages, and Tregs (Fig. 3C–I). Consistently, T helper cells were more T_H_1-like (Fig. 3H). Again, the Western diet + CCl_4_ MASH model demonstrated strain-specific liver immune changes. Hepatic CD8+ T cells were increased in FVB/N mice, but not in BALB/c or C57BL/6 mice (Fig. 3C). The decrease of CD4^+^ T cells was only observed in BALB/c mice (Fig. 3C). The B cells loss was greater in C57BL/6 mice than BALB/c mice, but could not be found in FVB/N mice (Fig. 3C). The MAIT cell expansion was observed in C57BL/6 and FVB/N but not in BALB/c mice, and the increase was much pronounced in FVB/N mice (Fig. 3D). The decrease of γδT cells was only found in FVB/n mice, which had the highest frequency of γδT cells (Fig. 3D). A significant increase in hepatic NK cells was only found in BALB/c mice (Fig. 3E). A drastic decrease in ILC1s occurred in FVB/n mice, but no change in ILC1 was found in BALB/c or C57BL/6 mice (Fig. 3E). Unlike BALB/c or C57BL/6 mice, FVB/N mice did not exhibit an increase in ILC2s (Fig. 3E), whereas the ILC3 increase was only found in FVB/N mice (Fig. 3E). The accumulation of hepatic myeloid cells was found in all mouse strains in the Western diet + CCl_4_ model, and BALB/c mice showed both the highest baseline and upregulation of neutrophils and macrophages (Fig. 3F). Importantly, many immune cells, particularly γδT cells (Fig. 3D, Fig. 2C), ILC2s (Fig. 3E, Fig. 2D), and macrophages (Fig. 3F, Fig. 2E) showed similar changes in all strains in both MASH models.

### Different changes of hepatic immune cells in mouse strains bearing the same liver cancer

**Fig. 4:**
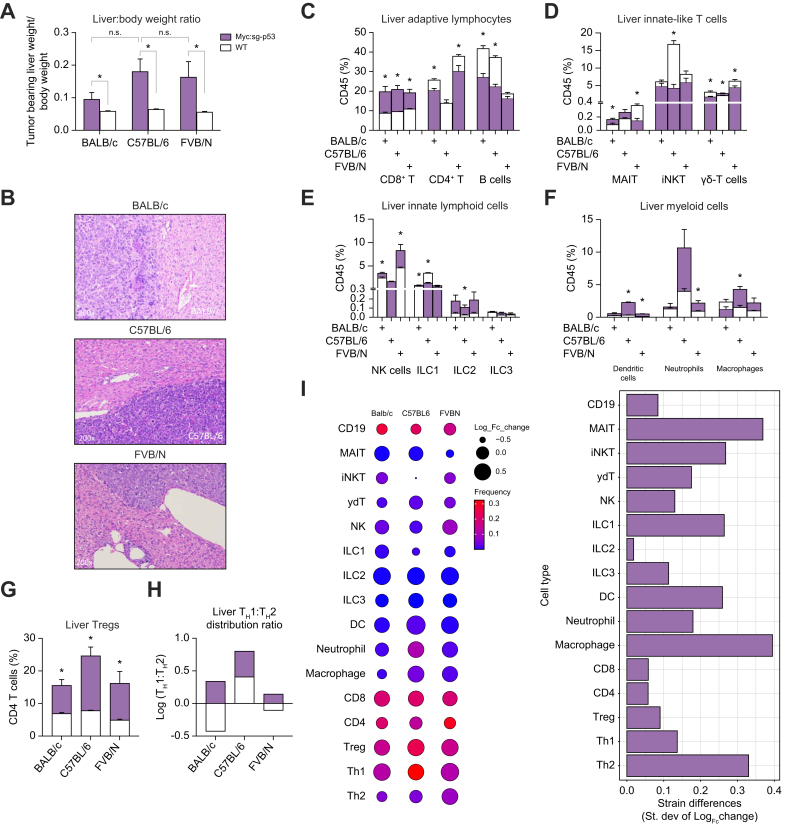
Liver immune cell changes in BALB/c, C57BL/6, and FVB/N mice bearing liver tumor. Female Mice with difference strain backgrounds were given hydrodynamic injection of either MYC/sg-p53 plasmids to induce liver cancer or empty plasmids as control. (A) Liver tumor burden was measured by ratio of tumor-bearing liver weight to total body weight. (B) The presence of liver cancer was confirmed by H&E staining. Immune cells prepared from MYC/sg-p53-bearing livers or control livers were analyzed by flow cytometry assay. The comparison between MYC/sg-p53 (vermilion) with control (gray) was performed in each liver immune subsets of the three mouse strains (C–H). The overall changes of various liver immune subsets by liver tumor from three mouse strains are shown (I). The size of circle represents the log_10_ transformed fold changes of each immune subset. The color gradient represents the relative frequences of each immune subset. The distribution of fold changes of each immune cells is also shown; n = 4 per group, two-way ANOVA with Bonferroni correction, ∗*p* <0.05. Tregs, regulatory T cells.

Next, we evaluated the immune cell alterations caused by the presence of liver tumors among mouse strains. To generate comparable liver tumors in mouse strains with different genetic backgrounds, the hydrodynamic oncogene delivery method was used, which has been well established to induce liver tumors independent of mouse strains. The combination of MYC oncogene with CRISPR-Cas9 (clustered regularly interspaced short palindromic repeats)-dependent TP53 knockout was chosen, as the induced HCC tumors were reported to show moderate immune infiltration.^11^^,^[14], [15], [16], [17] BALB/c, C57BL/6, and FVB/N mice were administered the MYC/sg-p53 plasmids or empty plasmids (lacking the MYC or the sg-RNA sequence) to control for the hydrodynamic injection technique. Mice were harvested 4 weeks post-injection (Fig. S4A). As reported, disseminated macroscopic liver tumors were found. Liver tumor burden was measured by calculating the widely used weight ratios between tumor-bearing livers *vs.* total body weight. Increased liver-to-body weight ratios were found in all mice injected with MYC/sg-p53 plasmids when compared with the control mice, regardless of mouse strains (Fig. 4A). The increase of liver-to-body weight ratio was similar between C57BL/6 and FVB/N mice, but trended less in BALB/c mice. The presence of neoplastic foci was confirmed by histology in each mouse (Fig. 4B).

The immune cell compositions were measured by taking into account cells isolated from tumor-bearing livers. It should be noted that because of the disseminated spread of liver tumors from our selected model, these liver immune cells contained tumor infiltrating cells as well. In the presence of tumor, liver immune cells continued to be dominated by T cells, followed by B cells (Fig. 4C, Fig. S2G–J). These tumors caused significant changes in the immune composition of hepatic tissues when compared with empty plasmid controls, many of which varied among mouse strains as found in our previous results. A consistent near two-fold increase of hepatic CD8^+^ T cells could be seen in all three strains of mice bearing the MYC/sg-p53 liver tumors (Fig. 4C). This result is consistent with previously published reports of moderate immunogenicity in the MYC/sg-p53 tumor model,^11^ in addition to the well-recognized antitumoral function of CD8^+^ T cells. MYC/sg-p53 liver tumors provoked significant decreases in hepatic CD4^+^ T cells and B cells, but these changes varied among mouse strains (Fig. 4C). The CD4^+^ T cell decrease was observed in BALB/c and FVB/N but not in C57BL/6 mice, whereas the reduction of B cells was seen among BALB/c and C57BL/6 but not in FVB/N mice. Great variation among mouse strains was also found in other immune subsets. A robust (∼50%) decrease of liver iNKT was found in tumor-bearing C57BL/6 mice, but the iNKT change was barely distinguishable in both BALB/c and FVB/N mice (Fig. 4D). Hepatic NK cells nearly doubled in FVB/N mice, but the NK cell increase was milder in BALB/c and negligible in C57BL/6 mice in the presence of tumor (Fig. 4E). Tumor-bearing C57BL/6 mice showed an immense increase in hepatic myeloid cells, especially neutrophils, but the myeloid cell increase was much milder in FVB/N mice and could not be observed in BALB/c mice (Fig. 4F).

Evaluation of liver CD4^+^ T cell subsets revealed a similar behavior among the MYC/sg-p53 liver tumor-bearing condition as seen in MASH mice. MYC/sg-p53 tumors increased hepatic FOXP3+ Tregs in all mouse strains (24.7% of CD4^+^ T cells in C57BL/6, 16.2% in FVB/N, and 15.6% in BALB/c), which were not significantly different from one another (Fig. 4G). The presence of tumor also increased the ratio of liver T_H_1 to T_H_2 cells across all mouse strains (log[T_H_1:T_H_2] = 0.80 in C57BL/6, 0.34 in BALB/c, 0.14 in FVB/N), and all mice strains presented a T_H_1 dominant CD4^+^ T helper status (Fig. 4H). This shift was mainly driven by a significant parallel increase in T_H_1 cells among all strains (logF[c] = 0.59 in FVB/N, 0.44 in BALB/c, 0.42 in C57BL/6).

The changes across liver immune cell when comparing MYC/sg-p53 tumor-bearing mice to tumor-free mice, as well as between mouse strains were evaluated (Fig. 4I, Fig. S5A–P). The most noticeable increases in the liver by MYC/sg-p53 tumors were seen among dendritic cells (logF[c] = 0.81 in C57BL/6, 0.59 in FVB/N, 0.30 in BALB/c) (Fig. 4I, Fig. S5K), followed by ILC2s (logF[c] = 0.64 in FVB/N, 0.61 in C57BL/6,0.60 in BALB/c) (Fig. 4I, Fig. S5J), CD8^+^ T cells (logF[c] = 0.35 in BALB/c, 0.34 in C57BL/6, 0.25 in FVB/N) (Fig. 4I, Fig. S5B), and neutrophils (logF[c] = 0.43 in C57BL/6, 0.37 in FVB/N, 0.09 in BALB/c) (Fig. 4I, Fig. S5L). Tumors caused negative shifts among strains in populations of liver iNKT cells (logF[c] = -0.59 in C57BL/6, -0.14 in FVB/N, -0.11 in BALB/c) (Fig. 4I, Fig. S5F), B cells (log F[c] = -0.22 in C57BL/6, -0.19 in BALB/c, -0.06 in FVB/N) (Fig. 4I, Fig. S5D), and CD4^+^ T cells (logF[c] = -0.10 in BALB/c, -0.10 in FVB/N) (Fig. 4I, Fig. S5C). Moreover, strains presented with opposite patterns of immune cell shifts among liver MAIT cells (logF[c] = 0.27 in BALB/c, 0.19 in C57BL/6, -0.40 in FVB/N) (Fig. 4I, Fig. S5E), ILC1s (logF[c] = 0.11 in BALB/c, -0.18 in FVB/N, -0.41 in C57BL/6) (Fig. 4I, Fig. S5I) and γδT cells (logF[c] = 0.10 in C57BL/6, -0.15 in FVBN, -0.23 in BALB/c) (Fig. 4I, Fig. S5G).

The changes in splenic immune cells under tumor-bearing conditions were examined (Fig. S4B–H, Fig. S5A′–O′). MYC/sg-p53 liver tumors had a limited impact on the levels of B cells, CD4^+^ T cells, and CD8^+^ T cells, which still covered >90% of CD45^+^ cells in the spleen (Fig. S4C). Similar to the liver, the presence of tumor caused a significant increase of myeloid cells in spleen (Fig. S4F). These changes in myeloid cells were less prominent in FVB/N mice, and not seen in BALB/c mice. The increase in splenic Tregs was also found among all mouse strains (Fig. S4G). Unlike in the liver, MYC/sg-p53 tumors caused a decrease in the ratios of T_H_1 to T_H_2 cells in BALB/c and C57BL/6 mice (log[T_H_1:T_H_2] = -0.18 and 0.21, respectively), but not in FVB/N spleens (Fig. S4H). The changes of most immune subsets varied between mouse strains, and shifts were often not consistent in proportion nor direction between liver and spleen microenvironments within the same mouse strain (Fig. S4B, Fig. S5A′–O′). When comparing splenic immune cell subsets altogether, neutrophils presented with the most prominent increase (logF[c] = 0.55 in FVB/N, 0.29 in C57BL/6, 0.10 in BALB/c) (Fig. S4B, Fig. S5K′), followed by macrophages (logF[c] = 0.16 in C57BL/6, 0.06 in FVB/N, 0.02 in BALB/c) (Fig. S4B, Fig. S5L′) and dendritic cells (logF[c] = 0.08 in FVB/N, 0.05 in BALB/c, 0.03 in C57BL/6) (Fig. S4B, Fig. S5J′). Decreases between all strains were also present among populations of MAIT cells (logF[c] = -0.46 in C57BL/6, -0.21 in BALB/c, -0.01 in FVB/N) (Fig. S4B, Fig. S5D′) and NK cells (logF[c] = -0.23 in BALB/c, -0.12 in FVB/N, -0.10 in C57BL/6) (Fig. S4B, Fig. S5G′). Differential immune cell shifts among strains were also observed among splenic CD8^+^ T cells and γδT cells, where these increased in C57BL/6 mice (logF[c] = 0.04 and 0.06, respectively) and decreased in both FVB/N (logF[c] = -0.07 and -0.05, respectively) and BALB/c mice (logF[c] = -0.02 and -0.08, respectively) (Fig. S4B, Fig. S5B′ and F′). Other differences in immune response to liver tumors were present among splenic iNKT cells, ILC1s, and ILC2s. No differences were encountered upon evaluation of B cells and ILC3s among strains.

Men are more likely to develop HCC and MASH-related HCC than women.^1^^,^^2^^,^^18^ Therefore, sex influence on the mouse liver immune system was studied using the MYC/sg-p53 HCC model. For each mouse strain, both male and female mice were assigned to the same batch of oncogene injection and immune cell profiling. A trend of bigger liver tumors was found in female mice (Fig. S6A), which was likely attributable to the relatively smaller female livers that could enhance hydrodynamic plasmid delivery. First, baseline liver immune cell levels were compared between male and female tumor-free control mice (wild-type group). Similar frequences were found in most liver immune cells (Fig. S6B–F), although some sex differences were observed. These differences were not consistent across the three mouse strains (Fig. S6B–F), showing that sex has no generalized impact on baseline liver immune populations. Next, HCC-induced liver immune cell changes were measured. Consistent with previous observations (Fig. 4C–G), a similar shift in liver immune landscape was found in tumor-bearing mice (Fig. S6B–F). Again, the sex differences of immune changes were minor and not shared by the three mouse strains (Fig. S6B–F). These results showed that sex had no generalizable impact on liver immune cells of either baseline or in this HCC mouse model.

In summary, our results highlight that the MYC/sg-p53 tumor model provoked substantial immune cell population changes in the liver, confirming its immunogenicity. As expected, immune alterations were much more prominent in the liver compared to spleen. Moreover, they presented with great variations among mouse strains. The shaping of the immune microenvironment following tumor progression is well documented. However, even though similar liver tumor burden was found between C57BL/6 and FVB/N mice, different tumor-induced immune cell changes were observed in these two strains. Together, our results suggest that using different mouse strains in research studies can greatly influence immune responses to tumor in the hepatic space.

### Immune change validation using published scRNA-seq datasets

We compared the flow cytometry-based immune profiling with published scRNA-seq datasets. The shift of liver immune landscape by tumor was analyzed in our recently reported scRNA-seq dataset,^19^ which was generated using sorted CD45^+^ cells from livers of C57BL/6 mice with or without orthotopic implantation of RIL-175 liver tumors. The dataset was processed using the Seurat package with the standard workflow. After several rounds of data filtration, the liver immune cells were separated into 16 clusters including six T cell subsets, B cells, NK cells, ILCs, three DC subsets, neutrophils, monocytes, and two macrophage subsets (Figs. S7A and B). The cluster annotations were validated with the expressions of characteristic cell markers (Fig. S7B). The change in immune cell distribution was visualized using UMAPs, with the increased myeloid subsets and decreased B cells being the most prominent alterations under tumor-bearing conditions (Fig. S7A). The scRNA-seq immune changes were quantified (Fig. S7C) and compared with flow cytometry results generated from C57BL/6 mice with or without MYC/sg-p53 tumor (Fig. S7D). Even with two different liver tumor models, the immune changes matched well between the scRNA-seq and flow cytometry data, both of which found prominent decreases in B cells and iNKT cells, as well as increases in Tregs, neutrophils, macrophages, and DCs (Figs. S7C and D). Although scRNA-seq did not observe the expansion of total CD8 T cells with tumors, a drastic upregulation of granzyme k (Gzmk)-producing CD8+ T subset was found, suggesting the accumulation of tumor-reactive CD8+ T cells.

The liver immune alternation by MASH was also analyzed in a published scRNA-seq dataset generated from C57BL/6 mice fed with Western or control diets.^20^ The dataset was similarly analyzed using the Seurat package, and 15 clusters were annotated covering the major immune cell types (Figs. S8A and B). It should be noted that the cell isolation method for this dataset included liver perfusion and collagenase digestion which enabled the recovery of Kupffer cells. Indeed, Kupffer cells were the most abundant immune cell type in control liver, and MASH caused a sharp drop of Kupffer cells as reported in the original study^20^ (Fig. S8A). In this dataset, the T cell sub-clustering was found with low FOXP3 detection. Following the original report, CD3+ cells were separated into general T cells and proliferating T cells. The scRNA-seq immune changes were calculated (Fig. S8C), and compared with flow cytometry results generated from C57BL/6 mice treated with MCD diet or Western diet + CCl_4_ (Figs. S8D and E). Despite the cell isolation method difference, many MASH-induced immune changes were preserved including the loss of B cells and increases in dendritic cells and macrophages (Figs. S8C–E). Together, our flow cytometry-based immune profiling can be validated by published scRNA-seq datasets.

### Cross-species comparison of MASH-induced liver immune changes between mice and human

**Fig. 5:**
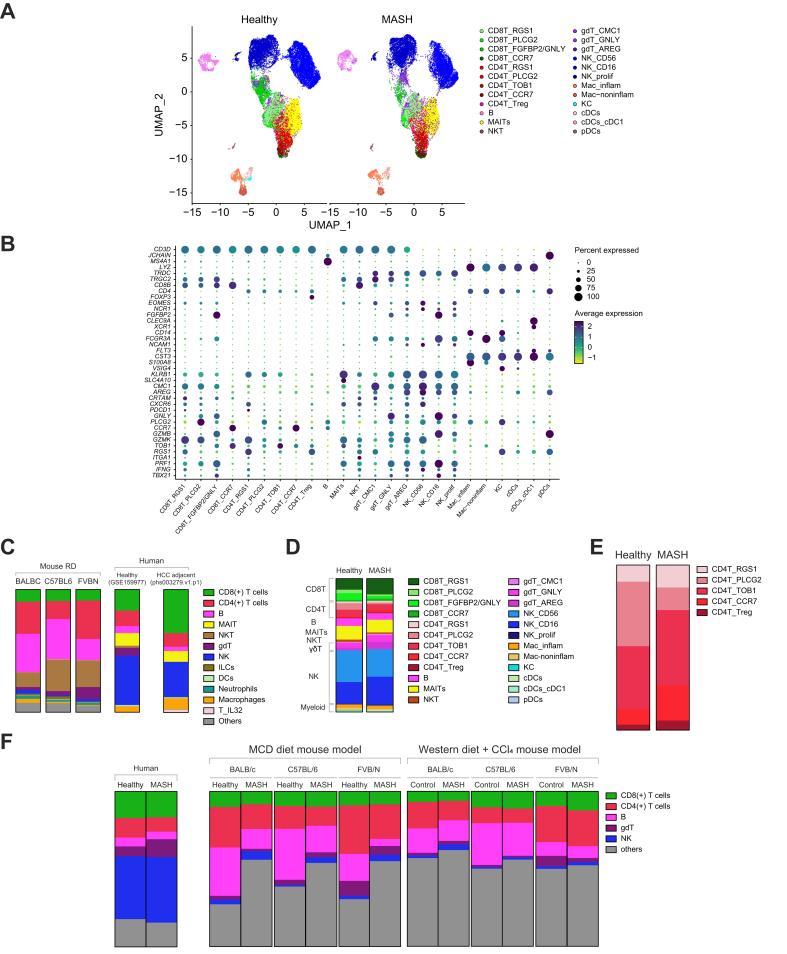
Cross-species comparison of liver immune changes by MASH between mice and humans. The published human scRNA-seq dataset GSE159977 of CD45+ cells from either MASH or healthy livers were processed using Seurat (5.1.0). (A) shows the UMAP split based on healthy or MASH. (B) shows the dot plot of marker genes for each annotated cell clusters. (C) Liver CD45^+^ cell compositions were measured in naïve BALB/c, C57BL/6, and FVB/N strains by flow cytometry as described in Fig. 1A. CD45^+^ cell composition of healthy human liver or human HCC adjacent liver tissues were calculated based on the scRNA-seq datasets of GSE159977 or phs003279.v1.p1, respectively. (D,E) CD45^+^ or CD4^+^ T cell compositions of MASH or healthy human livers were calculated from GSE159977. (F) In total liver CD45^+^ cells, the frequencies of shared major liver immune subsets between mice and human, including CD8+ T cell, CD4+ T cell, B cells, γδT cells and NK cells, were calculated in MASH or healthy human livers (GSE159977) and MASH or control mice of three strains under either MCD diet or Western + CCl_4_ diet MASH model. CCl_4_, carbon tetrachloride; HCC, hepatocellular carcinoma; MASH, metabolic dysfunction-associated steatohepatitis; MAIT cells, mucosal-associated invariant T cells; MCD diet, methionine- and choline-deficient diet; NK, natural killer; UMAP, Uniform Manifold Approximation and Projection.

Cross-species comparison of liver immune cells between mice and human was performed by analyzing a published scRNA-seq dataset (GSE159977) from Knolle’s group^12^ which included both histologically confirmed human MASH and healthy liver samples. Referencing the original clustering of T cells, we annotated the total CD45^+^ cells and separated them into four clusters of CD8^+^ T cells (RGS^+^CD8^+^ T cells, PLCG2^+^CD8^+^ T cells, FGFBP2/GNLY^+^CD8^+^ T cells, and CCR7^+^CD8^+^ T cells), five clusters of CD4^+^ T cells (RGS1^+^CD4^+^ T cells, PLCG2^+^CD4^+^ T cells, TOB1^+^CD4^+^ T cells, CCR7^+^CD4^+^ T cells, and Tregs), MAIT cells, natural killer T cells (NKT cells), three clusters of γδT cells (CMC1^+^ γδT cells, GNLY^+^ γδT cells, and AREG^+^ γδT cells), B cells, three clusters of NK cells (CD56^+^NK cells, CD16^+^NK cells, and proliferating NK cells), two clusters of macrophages (inflammatory macrophages and noninflammatory macrophages), Kupffer cells, and three clusters of dendritic cells (cDCs, cDC1, and pDCs) (Fig. 5A and B).

The baseline liver immune landscape was compared between mice and human. The most obvious cross-species difference is the dominance of NK cells in healthy human livers (Fig. 5C). Consistent with previous reports, human livers showed high MAITs but low NKT cells,^21^ which is opposite of that seen in the mouse. In human livers, the adaptive lymphocytes still presented as major immune subsets. Compared with NK and ILTCs, the levels of adaptive lymphocytes were relatively closer between mouse and human, but human livers had more CD8^+^ T cells than CD4^+^ T cells, the opposite of which was observed in the three mouse strains (Fig. 5C). The ratio between CD8^+^ T and CD4^+^ T cells in C57BL/6 mice was closer to human livers compared with the other two mouse strains. Human liver B cell frequencies were much lower compared with mice, and FVB/N mice had the lowest liver B cells amongst the three strains (Fig. 5C). These cross-species differences were preserved at the level of individual samples (Figs. S9A and B). As expected, the human samples showed much greater inter-sample variation than the three mouse strains (Fig. S9B). Furthermore, the mouse–human liver immune differences in this small cohort could be repeated in our reported dataset^19^ of non-tumor liver samples from eight patients with HCC (Fig. 5C), suggesting that the cross-species immune differences can be generalized.

Next, human liver immune changes with MASH were studied. As previously reported, MASH caused the expansion of liver RGS^+^CD8^+^ T cells (Fig. 5D), which resembled the auto-aggressive CXCR6^+^ CD8T cells.^12^ Interestingly, the level of total liver CD8^+^ T cells minimally changed in the patients with MASH (Fig. 5D), which is consistent with our mouse results (Fig. 2B, Fig. 3C). The decrease of total liver CD4^+^ T cells and B cells in MASH mice could be seen in this human dataset (Fig. 5D). Furthermore, MASH-induced Treg increases in mice (Fig. 2F, Fig. 3G) also repeated in patient MASH livers (Fig. 5E). These results suggest that mouse and human can share similar changes in adaptive immunity in the context of MASH. Unlike adaptive lymphocytes, the changes of ILTCs and innate lymphocytes were quite different (even opposite) between mice and humans. A decrease in liver MAIT cells was observed in patients with MASH (Fig. 5D), in contrast with their often increase in MASH mice (Fig. 2C, Fig. 3D). The low abundance and high variability of human liver NKT cells made it difficult to compare its regulation by MASH in the dataset (Fig. S9C). Patients with MASH showed an increase of liver γδT cells (Fig. 5D), whereas γδT cells were often found to be decreased in MASH mice (Fig. 2C, Fig. 3D). Although total liver NK cell frequencies did not change much, the proportion of CD16^+^ NK cells showed an increase in patients with MASH (Fig. 5D). Unlike in humans, an increase of total liver NK cells was often found in MASH mice (Fig. 2D, Fig. 3E). We did not further study myeloid cell regulation by MASH as they are sensitive to isolation methods and low levels of myeloid cells were found in this dataset (Fig. S9C). The immune changes were also measured in each patient, and excluded the possibility that the changes were caused by a single patient (Figs. S9C and D). It should be noted that considering the small sample size and high inter-sample variation in the human data, it is striking to see that many mouse MASH immune changes were still seen in this cohort.

We next tested which mouse strain could be the best to mimic the human MASH immune cell changes. Majored lymphocyte subsets, including CD8^+^ T, CD4^+^ T, B, γδT, and NK cells, were compared, as both myeloid and rare cell types are susceptible to influence of sample preparation. Human MASH livers showed reduced CD4^+^ T cells and B cells but increased γδT cells (Fig. 5F). Unsurprisingly, no tested mouse strain could completely mimic the human MASH immune changes (Fig. 5F). The reduction of CD4^+^ T cells and B cells, major adaptive lymphocytes, could be repeated in mice depending on both mouse strain and MASH model. In the MCD diet model, both BALB/c and FVB/N mice showed drops in CD4^+^ T cells (Fig. 5F). In the Western diet + CCl_4_ model, the loss of CD4^+^ T cells was only found in BALB/c mice (Fig. 5F), suggesting that BALB/c mice have an advantage to study MASH-reduced CD4^+^ T cells. The MCD diet model caused B cell reduction in all the three mouse strains, but mainly C57BL/6 mice showed B cell loss in the Western + CCl_4_ MASH model (Fig. 5F), indicating that C57BL/6 mice are more suitable to study B cell change in MASH. None of the three mouse strains in either MASH model could repeat the expansion of γδT cells in human MASH livers (Fig. 5F).

Together, our results support that despite major cross-species immune differences, mice are still valuable to recapitulate certain aspect of human immune regulations, and choosing mouse strains can potentially facilitate the investigation. The major immune differences among humans and the three mouse strains under different conditions were summarized in Table 1.

**Table 1:** Summary of major liver immune cell levels and changes in mice and humans under conditions of healthy individuals, MASH, and HCC.

Species and strain	Healthy	MASH		HCC (MYC/sg-p53 model)
		MCD diet	Western diet + CCl_4_	
BALB/c mouse	CD8^+^T (+), CD4^+^T (+++), B (++++), NKT (+)	CD4^+^T (↓↓), Treg (↑↑), B (↓↓↓),	CD4^+^T (↓↓), Treg (↑), NKT (↓↓↓)	CD8^+^T (↑↑), CD4^+^T (↓), Treg (↑↑↑), B (↓↓), NKT (↓↓)
C57BL/6 mouse	CD8^+^T (+), CD4^+^T (++), B (++++), NKT (+++)	B (↓↓), Treg (↑), NKT (↓↓)	B (↓), Treg (↑↑), NKT (↓↓↓)	CD8^+^T (↑↑), Treg (↑↑↑), B (↓↓), NKT (↓↓↓)
FVB/N mouse	CD8^+^T (+), CD4^+^T (++++), B (++), NKT (+++), γδT (+)	CD4^+^T (↓↓), Treg (↑↑), B (↓↓↓), NKT (↓↓), γδT (↓↓),	CD8^+^T (↑↑), Treg (↑↑), NKT (↓↓↓), γδT (↓↓)	CD8^+^T (↑↑), CD4^+^T (↓), Treg (↑↑↑), B (↓), NKT (↓↓), γδT (↓↓),
Human (GSE159977)	CD8^+^T (++), CD4^+^T (++), B (+), MAITs (++), γδT (+), NK (++++)	RGS1^+^CD8^+^T (↑↑), CD4^+^T (↓↓), Treg (↑↑), B (↓), γδT (↑↑), MAITs (↓)		N/A

Semiquantification of immune cell levels (of total CD45^+^ cells): 5∼10% (+), 10∼20% (++), 20∼30% (+++), >30% (++++); and immune changes: increase <20% (↑), increase 20∼100% (↑↑), increase >100% (↑↑↑), decrease <20% (↓), decrease 20∼50% (↓↓), decrease >50% (↓↓↓). CCl_4_, carbon tetrachloride; HCC, hepatocellular carcinoma; MASH, metabolic dysfunction-associated steatohepatitis.

## Discussion

Liver cancer is a leading cause of cancer-related deaths globally, often diagnosed at an unresectable stage with no effective treatment options.^1^ Liver cancer commonly arises from chronic inflammation such as viral hepatitis and MASH, and is considered to be a prototypical inflammation-driven cancer.^3^ MASH-associated HCC is increasing with the global obesity pandemic. Recent studies have demonstrated immunotherapy as a promising treatment for liver cancer, although the response rate is still low in patients.^22^ Understanding liver cancer immune regulations has become an important topic for developing better immune-based therapy approaches for liver cancer. Using mouse models, we and many other groups have discovered that both adaptive and innate immune systems are critical for the progression from MASH to liver cancer, and that MASH can impair immunotherapy against liver cancer.^9^^,^^13^^,^^23^^,^^24^ Mouse models are critical tools for the mechanistic study of antitumor immune regulations in liver cancer, but it should be noted that immune differences among mouse strains have long been recognized. Variable levels of immune subsets have been found in peripheral blood and hematopoietic organs across multiple strains of mice.^8^^,^^25^ Importantly, mouse strains can present differential immune functional states. For example, C57BL/6 mice are well known to preferentially develop T_H_1 immune responses, whereas BALB/c mice are prone for T_H_2 type immune responses. The liver harbors a large number of immune cells and is considered a lymphoid organ. However, it is still unclear how mouse strains affect the liver immune microenvironment and the responses to liver inflammatory diseases and ultimately liver cancer. In this study, we used high-dimensional flow cytometry-profiled liver immune subsets in three commonly used laboratory mouse strains side by in both non-pathologic and pathologic (MASH and liver cancer) states. Our results clearly demonstrate that in addition to the substantial baseline immune variations, the changes of immune landscape in response to MASH or liver cancer are quite different among mouse strains.

The mouse strain immune differences can potentially affect their usage to study human immune regulation. Major inter-species differences between mice and human were found in ILTCs and innate lymphocytes, particularly MAIT, NKT, and NK cells. The levels of adaptive lymphocytes were relatively similar between mice and human. Interesting, the shared MASH immune changes between mice and human were found to be limited to the adaptative lymphocytes including total CD4^+^ T loss, B cell loss and Treg cell increase. It is not surprising that mice can only mimic certain aspects of human immune regulation. Here our results support that depending on the human immune subsets of interest, different mouse strain can be more appropriate for mimicking human biology. BABL/c mice were more stable to recapture the CD4^+^ T loss based on our results in both MCD diet and Western + CCl_4_ MASH models, whereas C57BL/6 mice were more consistent to repeat the B cell reduction in MASH. It should be noted that the suggestion of BALB/c or C57BL/6 mice for mimicking human MASH CD4^+^ T or B cell change was based on a small MASH patient cohort, and humans have great inter-person/inter-race differences and vast genetic variation pool. Knowledge of mouse strain immune differences can provide more options to recapture the immune changes in various human populations. We also recognize many limitations of the cross-species comparison including the exclusion of myeloid cells because of the influence by sample preparation, small sample size, and lack of cell functional evaluation. Public human HCC datasets commonly use non-tumor adjacent liver as a control, which often had underlying liver disease and thus were not suitable for direct comparison with our mouse data. Besides mimicking the human immune system, mouse models are critical tools to test novel immune regulation mechanisms. Our study provides a resource of mouse strain-associated immune variations. Choosing the mouse strain with the desired baseline/change of the interested immune subsets can facilitate liver-related immune research.

Men are more likely to develop HCC and MASH-related HCC than women.^2^^,^^3^^,^^18^ Therefore, we directly studied the potential for sex to influence the liver immune system under both non-tumor and HCC-bearing conditions by comparing male and female mice side by side. A trend of higher tumor burden was found in female mice, but the effect was likely caused by enhanced hydrodynamic plasmid delivery in relatively smaller female mouse livers. In most liver immune cells, both baseline levels as well as the changes by MYC/sg-p53 HCC were found to be similar between female and male mice. Some sex-associated liver immune differences were found, but they were not shared across the three mouse strains. The results suggest that sex has no generalizable influence on mouse liver immune populations.

The data obtained from this study confirms that there are differences in immune cell composition within the spleens across the different strains of mice, as reported by previously published studies. For example, there was a higher percentage of splenic CD4^+^ T cells and a lower percentage of splenic neutrophils in BALB/c mice compared with C57BL/6 mice.^8^^,^^26^ However, new findings were reported in the immune cell composition of mouse livers. Interestingly, the balance of immune cell populations within mouse livers varied among BALB/c, C57BL/6, and FVB/N mice, establishing clear differences of their immune composition at baseline. The most impactful finding across these strains were present among liver lymphoid cells, where FVB/N strain mice presented with lower B cell and higher CD4^+^ T cell composition in comparison with C57BL/6 and BALB/c mice. Moreover, within hepatic CD4^+^ T cells, BALB/c strain mice presented with higher amounts in comparison to C57BL/6 mice, resembling the findings of splenic immune cells.

This study was not limited to the major immune cells commonly evaluated in research, as it also addressed populations of myeloid cell and innate-like lymphocytes that are relevant in the study of the hepatic microenvironment. For example, BALB/c mice exhibited an increased macrophage density when compared with C57BL/6 and FVB/N mice. However, it is important to note that the populations of macrophages defined in this study may not accurately represent the actual population present in living mice, as we followed protocols most commonly used for liver lymphocyte isolation, which do not favor macrophage recovery. However, FVB/N mice had an increased density of hepatic NK and γδT cells. Additionally, C57BL/6 mice presented with higher percentages of ILC1s, neutrophils, and iNKT cells. Regarding T helper and regulatory cells, T_H_1 and T_H_2 cells were predominantly present among C57BL/6 mice. However, when observing BALB/c mice alone, there is a higher amount of T_H_2 cells in comparison to T_H_1 cells in this strain exclusively, highlighting their previously established preference for a T_H_2 response.^7^

One of the novelties that this study presents is the characterization of immune cells among FVB/N strain mice. To date, there are limited studies that establish the immune microenvironment and/or response within these mice. Although it was clear that differences are present among strains, FVB/N mice presented an immune cell composition that resembled those present among BALB/c mice. Of note, one of the most prevalent populations within the livers of FVB/N mice was CD4^+^ T cells with more T_H_2 than T_H_1 cells, resembling the immune composition observed in BALB/c mice. This observation was also present among splenic immune cells in FVB/N strain mice. These findings suggest that more studies would be beneficial in establishing a model of immune response among these mice.

Taking into account that mouse strains have different responses to pathologies as reported in other studies,^25^^,^[27], [28], [29] we also considered the influence of liver diseases on immune cell shifts. First, we evaluated the influence of MASH on liver and systemic immunity among mice using MCD or Western diet + CCl_4_. We also compared immune microenvironment behaviors amongst mice of genetically induced HCC by hydrodynamic tail vein injection, using a MYC/sg-p53 plasmid system. There were differences in immune cell distributions in these models of disease compared with control groups, as well as differences between mouse strains. Even more interesting is that the degree and behavior of change of these populations when compared with controls also varied among strains. With these findings, it is important to highlight the influence of mouse strains on liver immunology, and these should be considered in the development of study designs of liver pathologies.

Our study has several limitations. MASH or liver tumors were investigated by using MCD/Western + CCl_4_ diets or the MYC/sg-p53 tumor model, respectively, at a single experimental endpoint. Although the design was sufficient to support that mouse stain differences indeed influenced liver immune environment under pathological conditions, the observed MASH or liver tumor-associated immune changes should be confirmed using additional models. In addition, our study was limited to immune cell phenotyping, and further functional studies are needed to better understand the relevance of these changes to disease progression, and their potential impact on treatments for MASH and liver cancer.

## Abbreviations

CCl_4_, carbon tetrachloride; HCC, hepatocellular carcinoma; ILCs, innate lymphoid cells; ILTCs, innate-like T cells; IFNγ, interferon γ; iNKT cells, invariant natural killer T cells; MAIT cells, mucosal-associated invariant T cells; MASH, metabolic dysfunction-associated steatohepatitis; MCD diet, methionine- and choline-deficient diet; NK cells, natural killer cells; Tregs, regulatory T cells; UMAP, Uniform Manifold Approximation and Projection; CRISPR, clustered regularly interspaced short palindromic repeats

## Financial support

TG was supported by the intramural research program of NIH, NCI (ZIA BC 011345).

## Authors’ contributions

Project concept: CM, TG. Performed experiments: PH, FHR, YM, XBZ, JQ. Analyzed data: PH, FHR, YM, XBZ, JQ. Contributed to data analysis PH, JQ, RT. Wrote the manuscript: FJR, CM. Contributed to manuscript editing: all authors.

## Data availability statement

All primary data associated with this study are present in the manuscript or the supplementary materials.

## Conflicts of interest

There are no conflicts of interest.

Please refer to the accompanying ICMJE disclosure forms for further details.
