# Supplementary material for: Hepatic immune environment differences among common mouse strains in models of MASH and liver cancer
Source: JHEP Rep. 2025 Mar 1;7(5):101380. doi: 10.1016/j.jhepr.2025.101380 (PMC12060451; doi:10.1016/j.jhepr.2025.101380)
Supplement: Multimedia component 1 [file mmc1.pdf]

Hepatic immune environment differences among common mouse strains in models of MASH and liver cancer

Patrick Huang, Francisco J. Rodriguez-Matos, Jonathan Qi, Rajiv Trehan, Yuta Myojin, Xiao Bin Zhu, Tim F. Greten, Chi Ma

Table of contents

Fig. S1……………………………………………………………………………………2

Fig. S2……………………………………………………………………………………3

Fig. S3……………………………………………………………………………………4

Fig. S4……………………………………………………………………………………5

Fig. S5……………………………………………………………………………………6

Fig. S6……………………………………………………………………………………7

Fig. S7……………………………………………………………………………………8

Fig. S8……………………………………………………………………………………9

Fig. S9……………………………………………………………………………………10

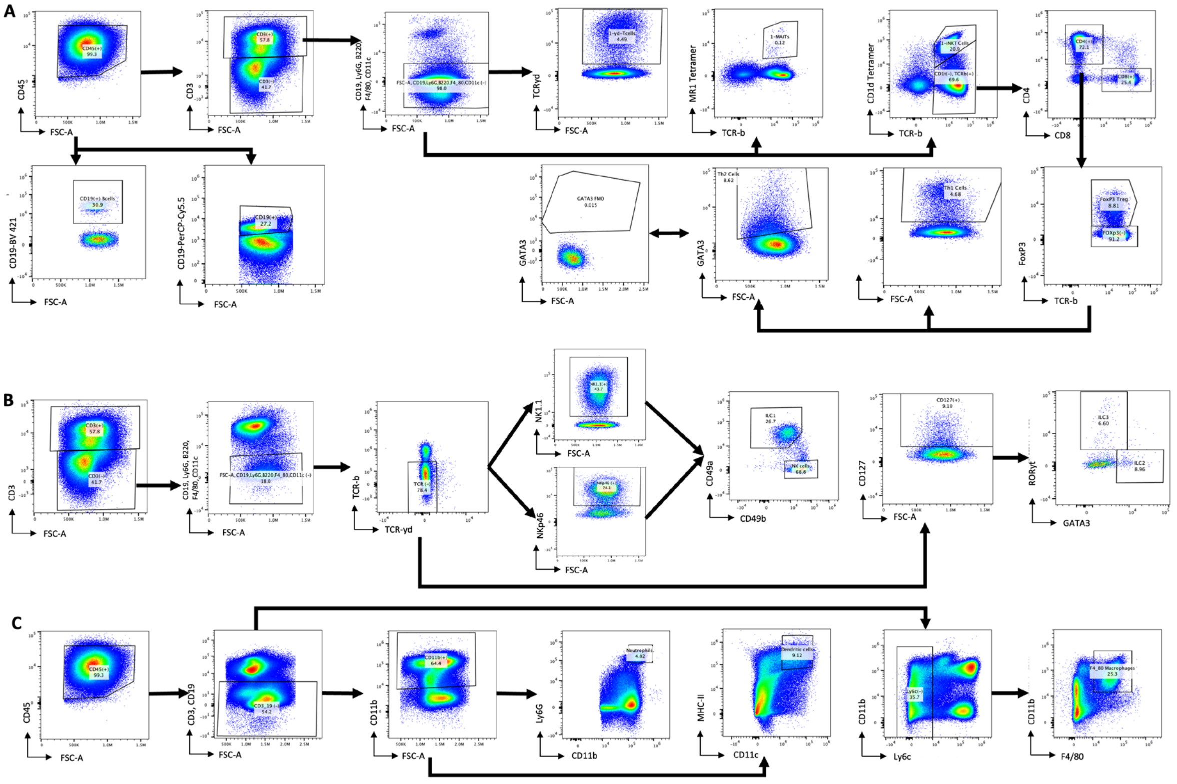

Fig. S1, Gating strategy.

(**A**) Gating for T and B subsets. CD8^{+}T cells were defined as CD45^{+}CD19^{-}B220^{-}Ly6G^{-}CD11c^{-}F4/80^{-}CD3^{+}TCRb^{+}CD8^{+}, CD4^{+} T cells as CD45^{+}CD19^{-}B220^{-}Ly6G^{-}CD11c^{-}F4/80^{-}CD3^{+}TCRb^{+}CD1d-teteramer^{-}CD4^{+}, iNKT as CD45^{+}CD19^{-}B220^{-}Ly6G^{-}CD11c^{-}F4/80^{-}CD3^{+}TCRb^{+}CD1d-teteramer^{+} , MAIT cells as CD45^{+}CD19^{-}B220^{-}Ly6G^{-}CD11c^{-}F4/80^{-}CD3^{+}TCRb^{+}MR1-teteramer^{+}, $\gamma\delta$–T cells as CD45^{+}CD19^{-}B220^{-}Ly6G^{-}CD11c^{-}F4/80^{-}CD3^{+} TCRdg^{+}, and B cells as CD45^{+}CD19^{+} . (**B**) Gating for innate lymphocytes. NK cells were defined as CD45^{+}CD19^{-}B220^{-}Ly6G^{-}CD11c^{-}F4/80^{-}CD3^{+}TCRb^{-} TCRdg^{-}NK1.1^{+} NK49a^{-}NK49b^{+} or CD45^{+}CD19^{-}B220^{-}Ly6G^{-}CD11c^{-}F4/80^{-}CD3^{+}TCRb^{-}TCRdg^{-}NKp46^{+}NK49a^{-}NK49b^{+}, ILC1 as CD45^{+}CD19^{-}B220^{-}Ly6G^{-}CD11c^{-}F4/80^{-}CD3^{+}TCRb^{-}TCRdg^{-}NK1.1^{+} NK49a^{+}NK49b^{-} , ILC2 as CD45^{+}CD19^{-}B220^{-}Ly6G^{-}CD11c^{-}F4/80^{-}CD3^{+}TCRb^{-} TCRdg^{-}CD127^{+}GATA3^{+} , and ILC3 as CD45^{+}CD19^{-}B220^{-}Ly6G^{-}CD11c^{-}F4/80^{-}CD3^{+}TCRb^{-} TCRdg^{-}CD127^{+}RORgt^{+}. (**C**) Gating for myeloid cells. Neutrophils were defined as CD45^{+}CD19^{-}CD3^{-}CD11b^{+}Ly6G^{+}, dendritic cells as CD45^{+}CD19^{-}CD3^{-}CD11c^{+}MHC^{hi}, and macrophages as CD45^{+}CD19^{-}CD3^{-}CD11b^{+}Ly6C^{-}F4/80^{+}.

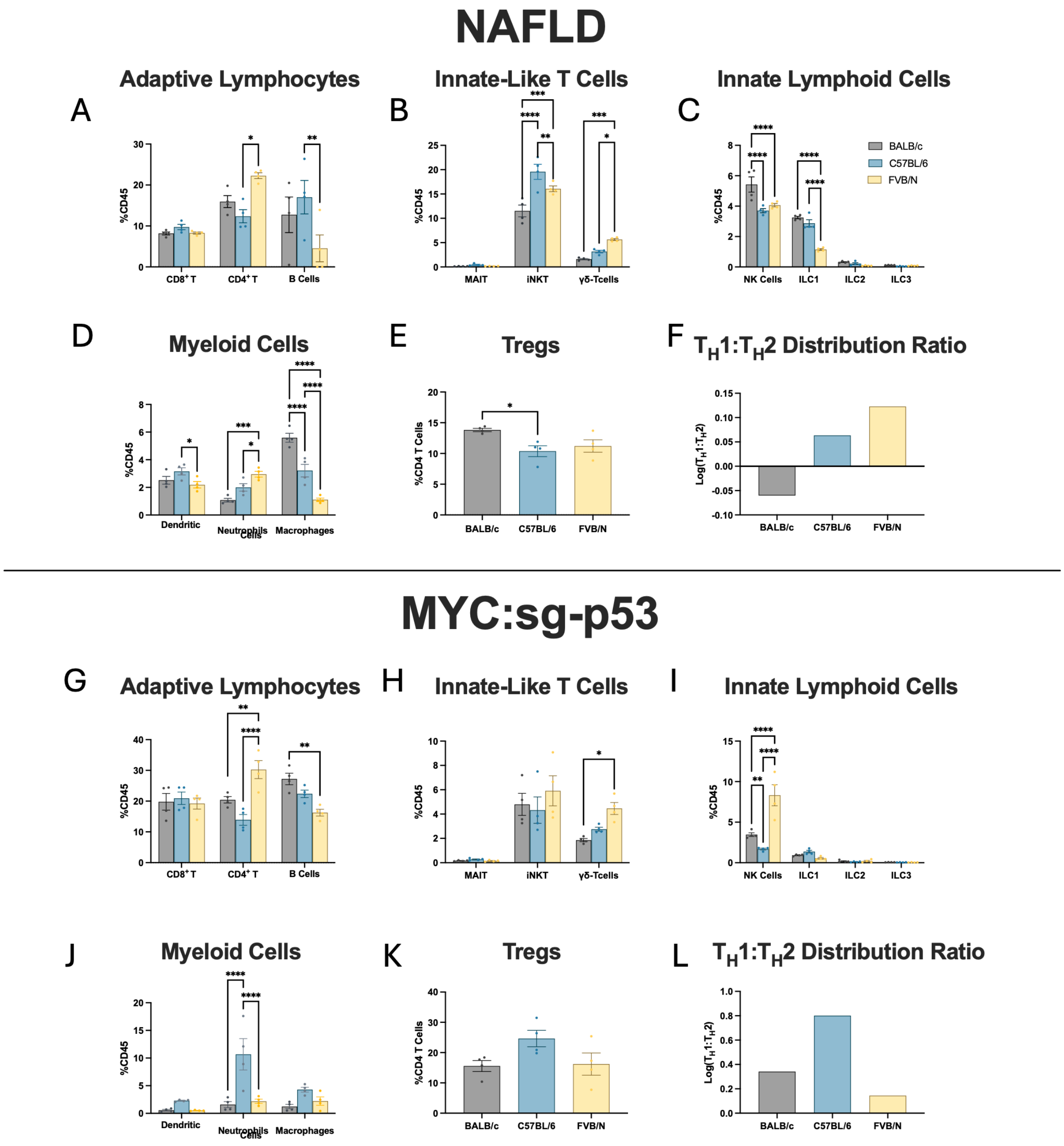

Fig. S2, The comparison among mouse strains in liver immune subsets under MASH or liver tumor.

(**A**-**F**) The comparison among BALB/c, C57BL/6 and FVB/N strains for each liver immune subsets after MCD diet feeding. (**G**-**K**) The comparison among BALB/c, C57BL/6 and FVB/N strains for each liver immune subsets from MYC;sg-TP53 bearing mice.

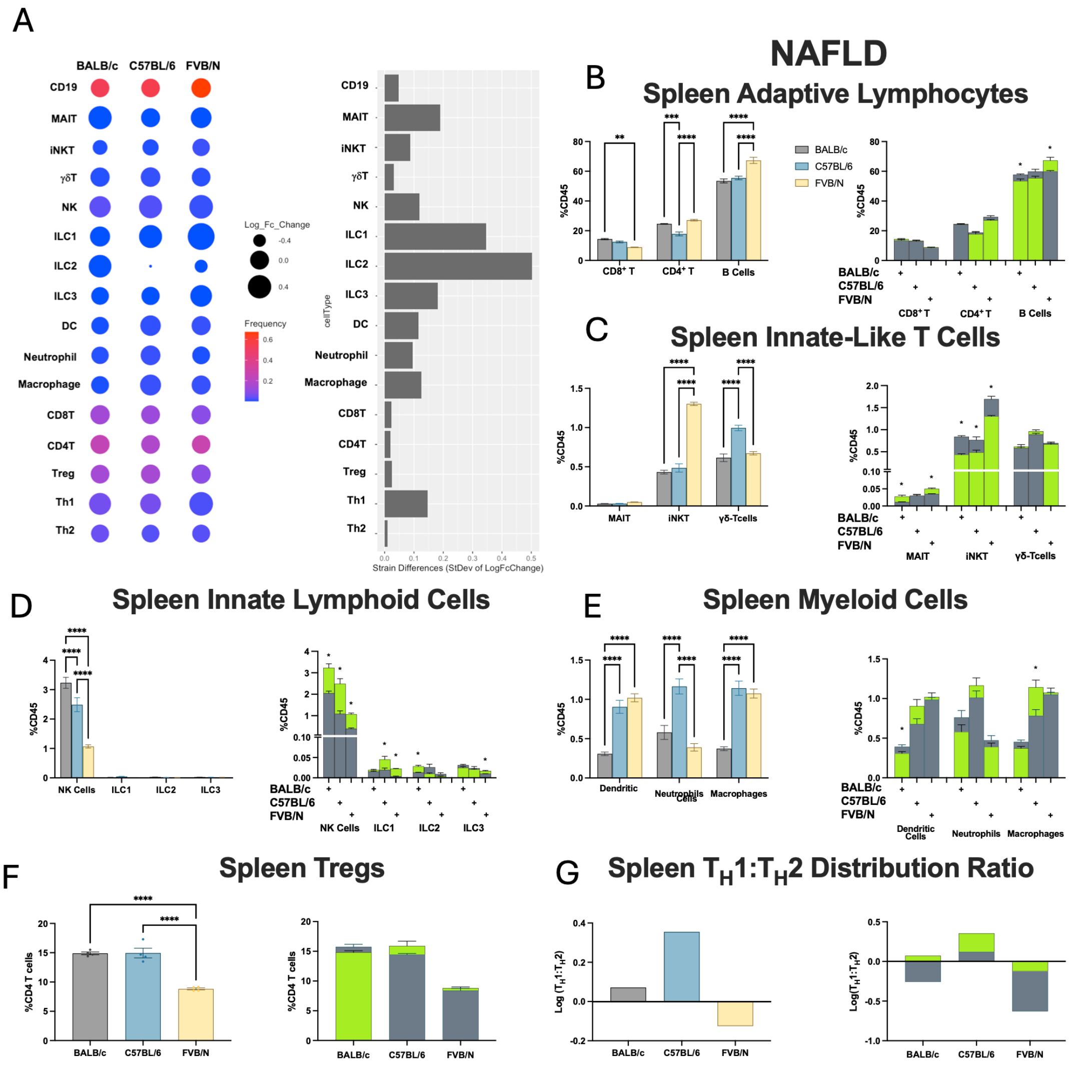

Fig. S3, Splenic immune cell changes in mouse strains with or without MASH. The overall changes of various spleen immune subsets by MCD diet feeding from three mouse strains were depicted in (**A**). The size of circle represents the log transformed fold changes of each immune subset. The color gradient represents the relative frequences of each immune subset. The distribution of fold changes of each immune cells was also shown. (**B**-**G**) The changes of each splenic immune subsets by MCD diet feeding are shown. For each panel the left part shows the comparison among mouse strains for each immune cell type measured under MASH condition. The right part focuses on the comparison between MCD and control diets.

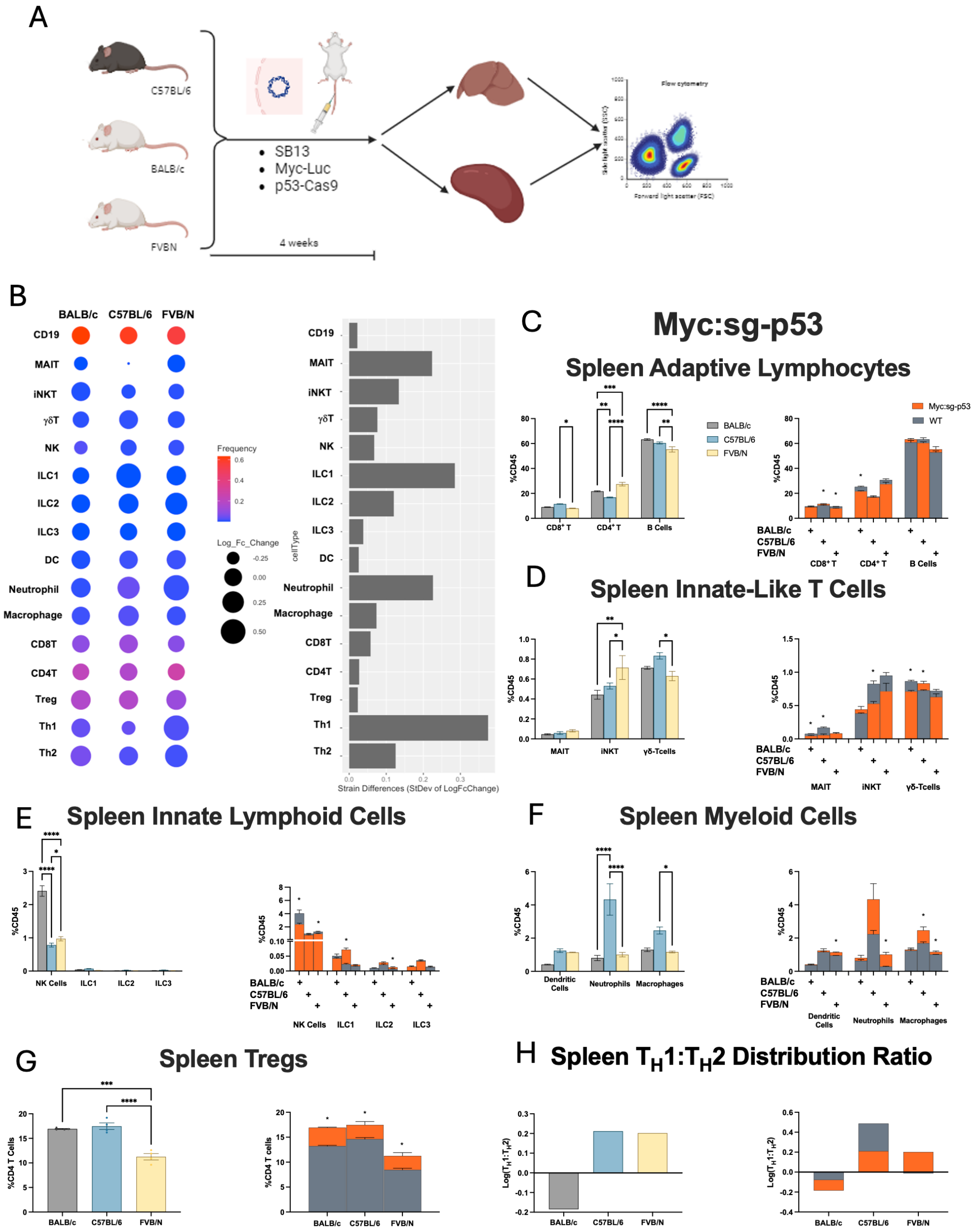

Fig. S4, Splenic immune cell changes in mouse strains with or without MYC:sg-p53 liver tumor.

(**A**) Experimental design. (**B**)The overall changes of splenic immune subsets in mice bearing MYC;sg-TP53 liver tumor. The size of circle represents the log transformed fold changes of each immune subset. The color gradient represents the relative frequences of each immune subset. The distribution of fold changes of each immune cells was also shown. (**C-H**) The changes of each spleen immune subsets by liver MYC;sg-TP53 tumor were shown. In each panel the left part shows the comparison among mouse strains for each immune cell type measured under tumor bearing condition. The right part focuses on the comparison between tumor and non-tumor groups.

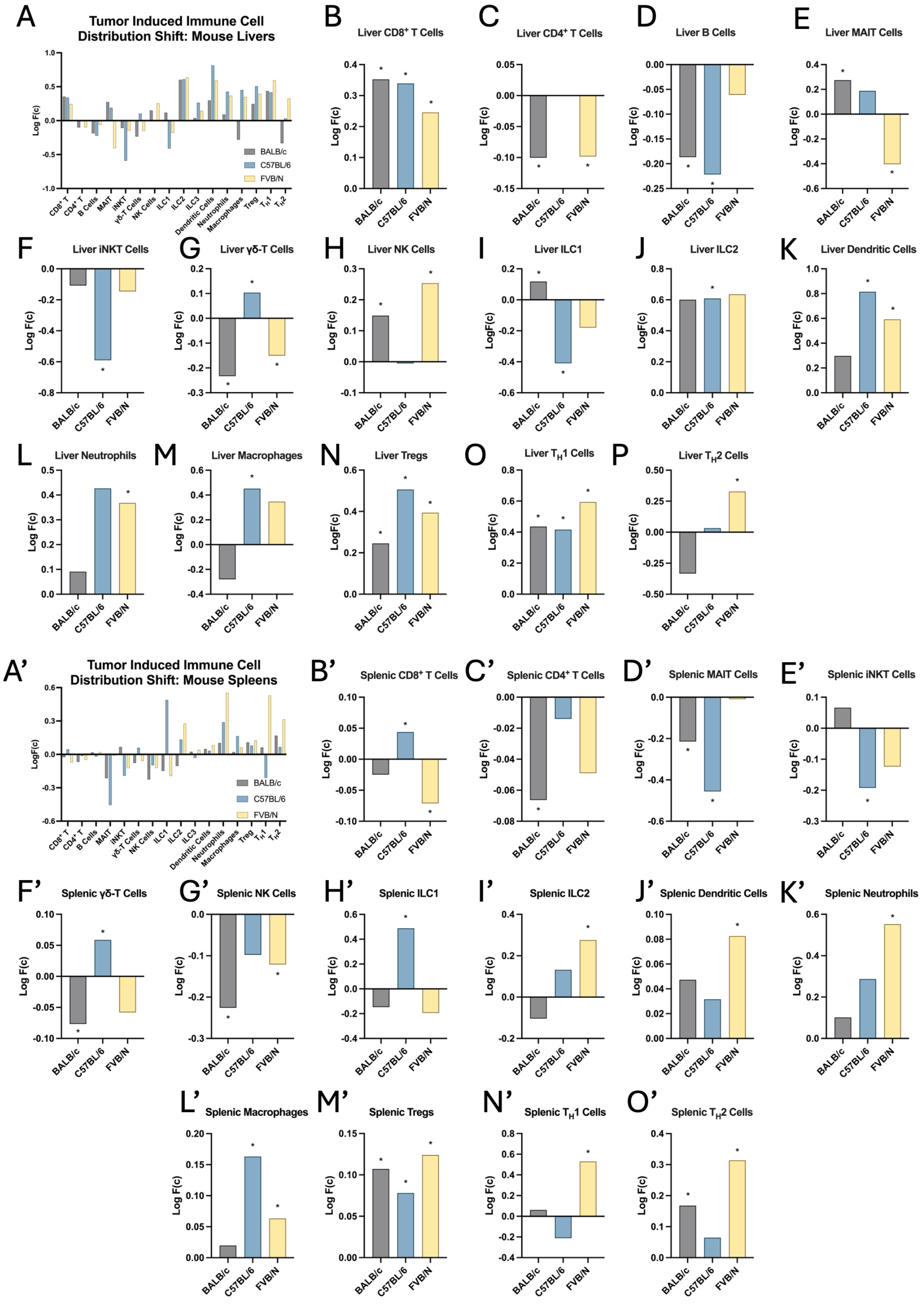

Fig. S5, Immune cell fold changes by MYC:sg-p53 liver tumor.

(**A**-**P**) The data of liver immune cell changed by MYC;sg-TP53 tumor from Fig.3I was shown in bar graph, either together (**A**) or separated by immune cell types (**B**-**P**). (**A’**-**O’**) The data of spleen immune cell changed by MYC;sg-TP53 tumor from Fig.S3B was shown in bar graph, either together (**A**) or separated by immune cell types (**B**-**P**).

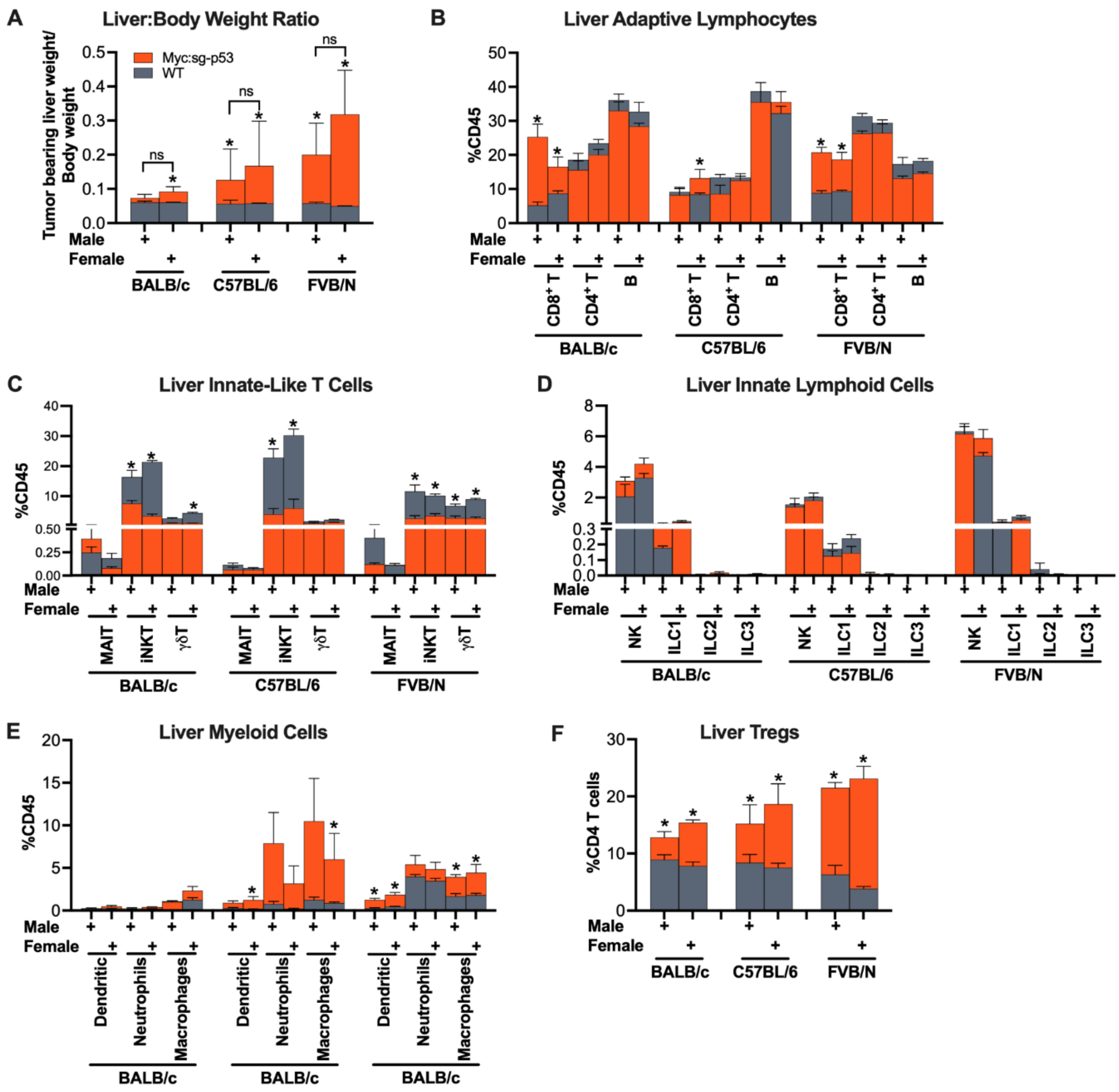

Fig. S6, Gender has no generalized impact on liver tumor-caused immune cell changes in BALB/c, C57BL/6 and FVB/N mice.

Both male and female mice with difference strain backgrounds were given hydrodynamic injection of either MYC/sg-p53 plasmids to induce liver cancer (Myc:sg-p53) or empty plasmids as control (WT). Bother genders of the same mouse strains were assigned to the same batch of injection and immune cell profiling. (A)Liver tumor burden was measured by ratio of tumor bearing liver weight to total body weight. (B-F) Immune cells prepared from MYC/sg-p53-bearing livers or control livers were analyzed by flow cytometry assay. n=4 per group, two-way ANOVA with Bonferroni correction, *p<0.05 indicates the significant change between Myc:sg-p53 (vermilion) with control (gray).

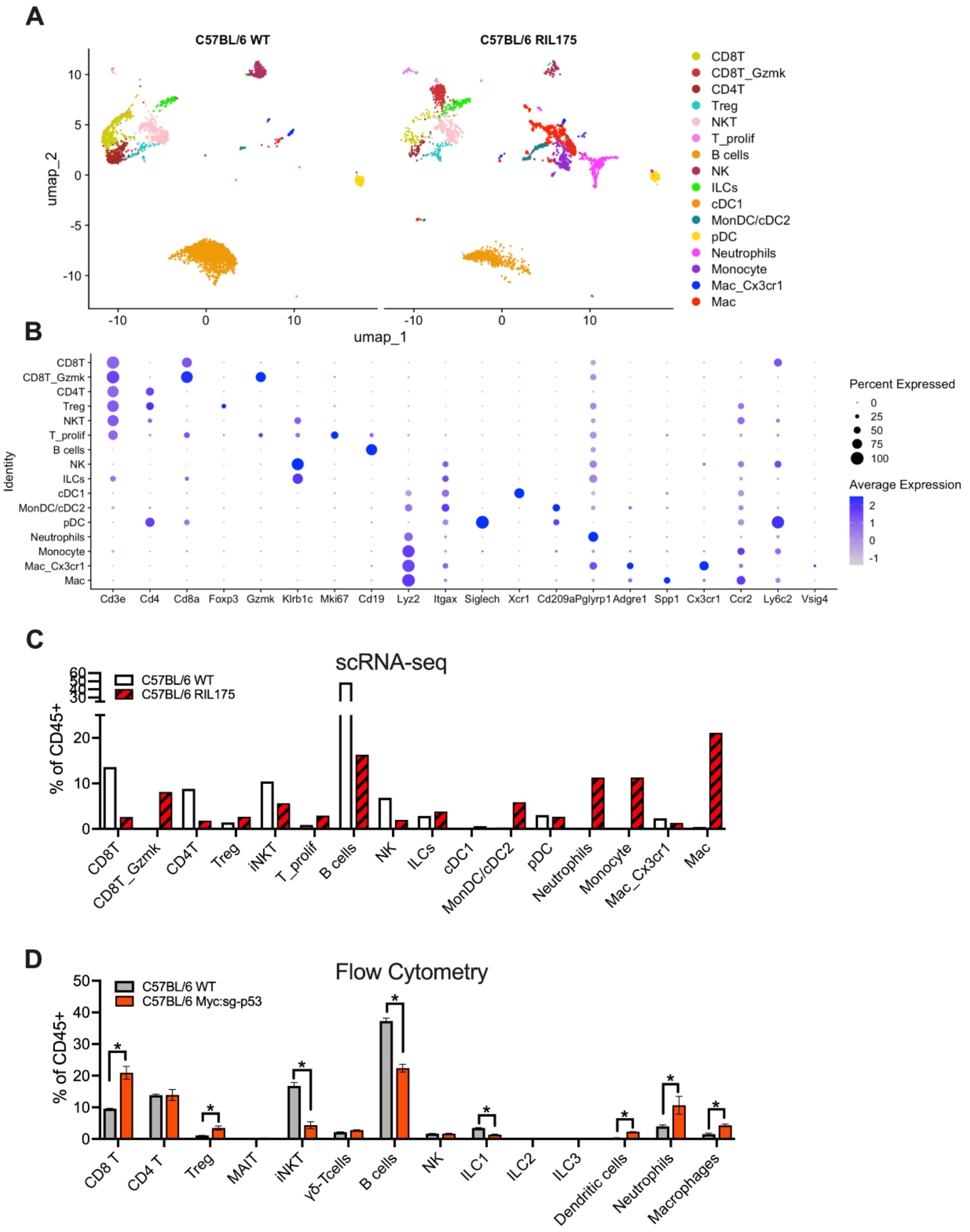

Fig. S7, Tumor-associated liver immune changes in published scRNA-seq data. The dataset GSE231712 with hepatic CD45+ cells from C57BL/6 mice bearing RIL-175 tumors was processed using the Seurat package (version 5.1.0) with standard workflow. (**A**)The cell changes between tumor free and tumor bearing conditions were visualized by UMAP. (**B**)The expressions of marker genes for each cell cluster were shown by dotplot. (**C**)The compositions of identified cells clusters were shown. (**D**) The compositions of various liver immune subsets measured by flow cytometry from C57BL/6 mice with or without MYC;sg-TP53 liver tumor.

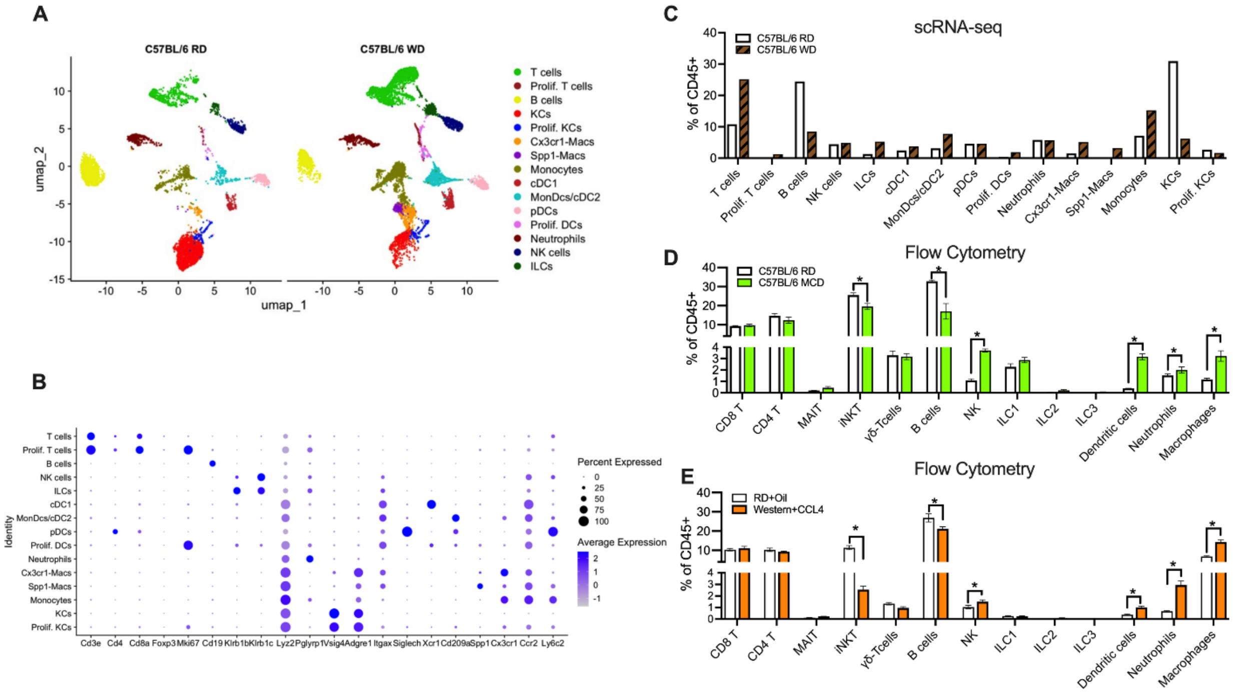

Fig. S8, MASH-induced liver immune changes in published scRNA-seq data.
The dataset GSE156059 with hepatic CD45+ cells from C57BL/6 mice fed with western diet was processed using the Seurat package (version 5.1.0) with standard workflow. (**A**)The cell changes between control and MASH induced by western diet were visualized by UMAP. (**B**)The expressions of marker genes for each cell cluster were shown by dotplot. (**C**)The compositions of identified cells clusters were shown. (**D**) The compositions of various liver immune subsets measured by flow cytometry from C57BL/6 mice fed with control or MCD diet. (**E**) The liver immune compositions measured by flow cytometry from C57BL/6 mice fed with control or western diet +CCL4 injections.

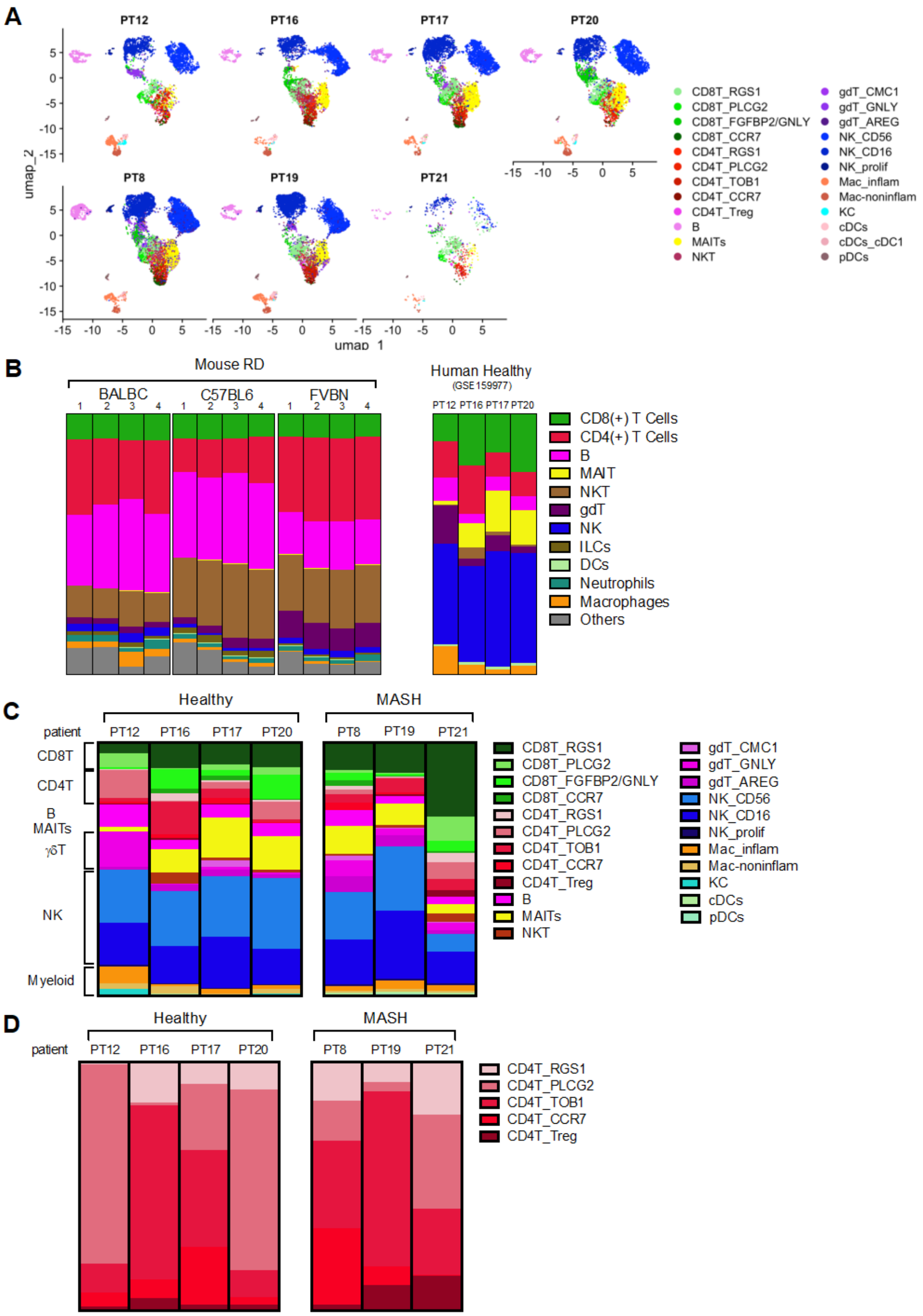

Fig. S9, Cross-species comparison of MASH-induced liver immune changes between mice and human.

The Human dataset GSE159977 of CD45$^+$ cells from MASH or healthy human livers was processed using the Seurat package (version 5.1.0) (**A**) shows UMAP of each individual sample. (**B**)Liver CD45$^+$ cell compositions in each naïve mouse of the 3 mouse strains or each healthy human liver. (**C**,**D**) Liver CD45$^+$ or CD4$^+$ T cell compositions of each samples of MASH or healthy human liver.
