# Supplementary material for: Hepatic immune environment differences among common mouse strains in models of MASH and liver cancer
Source: JHEP Rep. 2025 Mar 1;7(5):101380. doi: 10.1016/j.jhepr.2025.101380 (PMC12060451; doi:10.1016/j.jhepr.2025.101380)
Supplement: Multimedia component 2 [file mmc2.docx]

**JHEP Reports**

**CTAT methods**

Tables for a “Complete, Transparent, Accurate and Timely account” (CTAT) are now mandatory for all revised submissions. The aim is to enhance the reproducibility of methods.

- Only include the parts relevant to your study
- Refer to the CTAT in the main text as ‘Supplementary CTAT Table’
- Do not add subheadings
- Add as many rows as needed to include all information
- Only include one item per row

**If the CTAT form is not relevant to your study, please outline the reasons why:**

|  |
| --- |

- 1. **Antibodies**

| **Name** | **Citation** | **Supplier** | **Cat no.** | **Clone no.** |
| --- | --- | --- | --- | --- |
| CD45- BUV395 | PMID: 37635810 | BD Biosciences | 567451 | I3/2.3 |
| CD3- APC | PMID: 31269438 | BioLegend | 100236 | 17A2 |
| CD19- APC | PMID: 34161762 | BioLegend | 152410 | 1D3/CD19 |
| NKp46- FITC | PMID: 33290276 | BioLegend | 137606 | 29A1.4 |
| CD11b- BV421 | PMID: 30824325 | BioLegend | 101251 | M1/70 |
| F4/80- BUV496 | PMID: 21952799 | BD Biosciences | 750644 | T45-2342 |
| VSIG4- BV510 | PMID: 22366893 | BD Biosciences | 749505 | 17c9 |
| Ly6C- PE/Cy7 | PMID: 37179332 | BioLegend | 128018 | HK1.4 |
| Ly6G- AF700 | PMID: 33086061 | BioLegend | 127622 | 1A8 |
| CD11c- BUV737 | PMID: 38663398 | BD Biosciences | 612797 | HL3 |
| MHC-II- BV785 | PMID: 34875227 | BioLegend | 107645 | M5/114.15.2 |
| PD-L1- BV650 | PMID: 22351930 | BioLegend | 124336 | 10F.9G2 |
| CD3- BUV496 | PMID: 31632026 | BD Biosciences | 741117 | 17A2 |
| MHC-II- BV563 | PMID: 38625791 | BD Biosciences | 748846 | 34-5-3 |
| TCR-yd- BUV661 | PMID: 8105480 | BD Biosciences | 750410 | GL3 |
| TCRb- BUV737 | PMID: 38181094 | BD Biosciences | 612821 | H57-597 |
| NK1.1- BUV805 | PMID: 36991128 | BD Biosciences | 741926 | PK136 |
| Ly6G- BV421 | PMID: 36306422 | BioLegend | 127628 | 1A8 |
| B220- BV421 | PMID: 36932063 | BioLegend | 103240 | RA3-62B |
| F4/80- BV421 | PMID: 34986334 | BioLegend | 123132 | BM8 |
| CD11c- BV421 | PMID: 30824325 | BioLegend | 117330 | N418 |
| CD19- PerCP-Cy5.5 | PMID: 32165633 | BioLegend | 115534 | 6D5 |
| EOMES- eFluor450 | PMID: 36964178 | Invitrogen | 48-4875-82 | Dan11mag |
| NKp46- BV480 | PMID: 10092106 | BD Biosciences | 746264 | 29A1.4 |
| CD49b- BV510 | PMID: 32383173 | BD Biosciences | 740133 | HMa2 |
| CD4- BV570 | PMID: 30527911 | BioLegend | 100542 | RM4-5 |
| CD127- BV605 | PMID: 35732731 | BioLegend | 135041 | A7R34 |
| ST-2 (IL-33R)- BV645 | PMID: 36371464 | Invitrogen | 64-9335-82 | RMST2-2 |
| CD49a- BV711 | PMID: 39225100 | BD Biosciences | 564863 | Ha31/8 |
| KLRG1- BV750 | PMID: 37116491 | BD Biosciences | 746972 | 2F1 |
| CD8a- BV786 | PMID: 38702146 | BD Biosciences | 563332 | 53-6.7 |
| Ki67- AF532 | PMID: 33596419 | Invitrogen | 58-5698-82 | SolA15 |
| FOXP3- FITC | PMID: 26056145 | Invitrogen | 11-5773-82 | FJK-16s |
| GATA3- BB700 | PMID: 21358638 | BD Biosciences | 566643 | L50-823 |
| CD1d-Tetramer- PE | PMID: 29798856 | NIH Tetramer Core Facility | 63726 | N/A |
| RORyt- PE-CF594 | PMID: 38157853 | BD Biosciences | 562684 | Q31-378 |
| CD39- PE/Fire640 | PMID: 19877008 | BioLegend | 143818 | Duha59 |
| CD25- SparkYG 581 | PMID: 17620363 | BioLegend | 102074 | PC61 |
| T-bet- PE/Cy7 | PMID: 34847356 | BioLegend | 644824 | 4B10 |
| MR1 Tetramer- APC | PMID: 37595566 | NIH Tetramer Core Facility | 63725 | N/A |
| c-kit- APC/Fire 750 | PMID: 1378071 | BioLegend | 135140 | ACK2 |

- 1. **Organisms**

| **Name** | **Citation** | **Supplier** | **Strain** | **Sex** | **Age** | **Overall n number** |
| --- | --- | --- | --- | --- | --- | --- |
| Mus musculus | PMID: 29798856 | Charles River | C57BL/6 | M&F | 6-8 weeks old at experimental start point | 30 |
| Mus musculus | PMID: 29798856 | Charles River | BALB/c | M&F | 6-8 weeks old at experimental start point | 30 |
| Mus musculus | PMID: 26934227 | Charles River | FVB/N | M&F | 6-8 weeks old at experimental start point | 30 |

- 1. **Software**

| **Software name** | **Manufacturer** | **Version** |
| --- | --- | --- |
| FlowJo | FlowJo LLC | v10.9.0 |
| PRISM Graph Pad | GraphPad Software, Inc | v10.2.0 (355) |
| RStudio | Posit, PBC | version 2023.09.1+494 |
| Package ggplot2 | Hadley Wickham | version 3.5.0 |
| Package Seurat | Satija Lab | v5.1.0 |

- 1. **Other (*e.g*. drugs, proteins, vectors etc.)**

| Methionine Choline Deficient (MCD) diet | Research diets | Ref. A02082002BR |
| --- | --- | --- |
| Western Diet | Envigo | Ref. TD.120528 |
| Carbon tetrachloride(CCL4) | Sigma | Cat#289116 |
| D-fructose | Sigma | Cat#F0127 |
| D-glucose | Sigma | Cat#G8270 |
| pT3-EF1a-MYC | Addgene plasmid | #92046 |
| px330-sg-p53 | Provided by Dr. Amaia Lujambio | PMID: 32814112 |
| SB13 | Provided by Dr. Amaia Lujambio | PMID: 32814112 |

- 1. **Please provide the details of the corresponding methods author for the manuscript:**

| Chi Ma,  Thoracic and GI Malignancies Branch, National Cancer Institute,  Bldg10 RM3B44, 10 Center Dr. Bethesda, MD, 20892 |
| --- |
